# Genome‐wide dissection of AP2/ERF and HSP90 gene families in five legumes and expression profiles in chickpea and pigeonpea

**DOI:** 10.1111/pbi.12520

**Published:** 2016-01-23

**Authors:** Gaurav Agarwal, Vanika Garg, Himabindu Kudapa, Dadakhalandar Doddamani, Lekha T. Pazhamala, Aamir W. Khan, Mahendar Thudi, Suk‐Ha Lee, Rajeev K. Varshney

**Affiliations:** ^1^ International Crops Research Institute for the Semi‐Arid Tropics (ICRISAT)HyderabadIndia; ^2^ Department of Plant ScienceResearch Institute for Agriculture and Life SciencesSeoul National UniversitySeoulKorea; ^3^ Plant Genomics and Breeding InstituteSeoul National UniversitySeoulKorea; ^4^ School of Plant BiologyInstitute of AgricultureThe University of Western AustraliaCrawleyWAAustralia

**Keywords:** AP2/ERF, HSP90, chickpea, pigeonpea

## Abstract

APETALA2/ethylene response factor (AP2/ERF) and heat‐shock protein 90 (HSP90) are two significant classes of transcription factor and molecular chaperone proteins which are known to be implicated under abiotic and biotic stresses. Comprehensive survey identified a total of 147 AP2/ERF genes in chickpea, 176 in pigeonpea, 131 in Medicago, 179 in common bean and 140 in Lotus, whereas the number of HSP90 genes ranged from 5 to 7 in five legumes. Sequence alignment and phylogenetic analyses distinguished AP2, ERF, DREB, RAV and soloist proteins, while HSP90 proteins segregated on the basis of their cellular localization. Deeper insights into the gene structure allowed ERF proteins to be classified into AP2s based on DNA‐binding domains, intron arrangements and phylogenetic grouping. RNA‐seq and quantitative real‐time PCR (qRT‐PCR) analyses in heat‐stressed chickpea as well as Fusarium wilt (FW)‐ and sterility mosaic disease (SMD)‐stressed pigeonpea provided insights into the modus operandi of AP2/ERF and HSP90 genes. This study identified potential candidate genes in response to heat stress in chickpea while for FW and SMD stresses in pigeonpea. For instance, two DREB genes (Ca_02170 and Ca_16631) and three HSP90 genes (Ca_23016, Ca_09743 and Ca_25602) in chickpea can be targeted as potential candidate genes. Similarly, in pigeonpea, a HSP90 gene, C.cajan_27949, was highly responsive to SMD in the resistant genotype ICPL 20096, can be recommended for further functional validation. Also, two DREB genes, C.cajan_41905 and C.cajan_41951, were identified as leads for further investigation in response to FW stress in pigeonpea.

## Introduction

Tropical food legumes like chickpea (*Cicer arietinum*), pigeonpea (*Cajanus cajan*) and common bean (*Phaseolus vulgaris*) play an important role in reducing poverty, improving human health and nutrition, besides leading to ecosystem resilience. Globally 71.7 million tons of pulses (chickpea, pigeonpea and beans) were produced during 2013 and consumed in various forms (http://www.fao.org/docrep/019/i3751e/i3751e.pdf). Temperate legume species like Medicago (*Medicago truncatula)* and Lotus (*Lotus japonicus)* are considered as model legumes for genomics and physiological studies. Moreover, their syntenic relationship with other related legume crops could be helpful in better understanding the gene families relevant to both biotic and abiotic stress tolerances (Young and Udvardi, 2009). Legume crops of economic importance such as chickpea, common bean and pigeonpea have not witnessed expected increase in production and productivity in recent past (Varshney *et al*., 2010). Abiotic stresses such as drought, heat, cold, high salinity and biotic stresses such as Fusarium wilt (FW), Ascochyta blight and sterility mosaic disease (SMD) have been reported to reduce the average yield drastically in these crops.

In addition to conventional breeding strategies, several omics technologies are being deployed for improvement of these crops. However, mining and characterization of stress‐responsive genes will facilitate their use in crop improvement programmes. Previously, characterization of various stress‐responsive genes or gene families has mostly been limited to model crops such as Arabidopsis (*Arabidopsis thaliana*), rice (*Oryza sativa*) and maize (*Zea mays*). However, in the case of chickpea, comprehensive resources for gene discovery were developed in response to drought and salinity stresses (Varshney *et al*., 2009). In addition, comprehensive transcriptome assemblies were also developed (Hiremath *et al*., 2011; Kudapa *et al*., 2014). Similarly, in the case of pigeonpea, candidate genes associated with FW and SMD were mined (Raju *et al*., 2010) and transcriptome assembly was developed using Sanger sequencing as well as next‐generation sequencing (NGS) technologies (Kudapa *et al*., 2012).

Among stress‐responsive gene families, APETALA2/ethylene response factor (AP2/ERF) superfamily and heat‐shock protein 90 (HSP90) family are important, as they not only regulate responses against various biotic and abiotic stresses in plants, but also play an important role in various developmental processes (Mizoi *et al*., 2012; Wessler, 2005). Until recently, lack of information on legume genomes restricted the genome‐wide survey of genes implicated in biotic and abiotic stresses. In recent years, genome sequences of chickpea (Varshney *et al*., 2013), pigeonpea (Varshney *et al*., 2012), common bean (Schmutz *et al*., 2014), Medicago (Young *et al*., 2011) and Lotus (Sato *et al*., 2008) have become available.

Heat stress in chickpea and FW and SMD in pigeonpea are major yield reducers in these crops. AP2/ERF and HSP90 genes are known to be implicated in both biotic and abiotic stresses and AP2/ERF family TFs were found involved both in developmental regulation and stress response in plants. The induction of HSP expression against high temperatures is one of the best‐characterized responses. HSP90 chaperones are constitutively expressed in most organisms under normal conditions, while their expression increases significantly under stress. HSP90s play a vital role in plant development, stress response and disease resistance (Lindquist and Jarosz, 2010; Sangster and Queitsch, 2005; Takahashi *et al*., 2003; Xu *et al*., 2012). Recent studies have also shown relation between heat stress‐induced gene expression and DREB2A gene (Sato *et al*., 2014).

In view of above, we identified AP2/ERF and HSP90 gene family in chickpea, pigeonpea, common bean, Medicago and Lotus in the present study. We also conducted phylogenetic, syntenic, evolutionary studies, apart from their gene and protein structure analysis. In addition, expression profiling of these genes using RNA‐seq data of heat stress in chickpea and FW and SMD stress in pigeonpea has also been performed. Furthermore, quantitative real‐time PCR (qRT‐PCR) validation of selected AP2/ERF and all HSP90 genes in different tissues of contrasting genotypes for heat stress in chickpea and pathogen stress in pigeonpea was examined to confirm the expression patterns of the selected genes.

## Results and discussion

### Identification of AP2/ERF transcription factor superfamily genes

**Figure 1:**
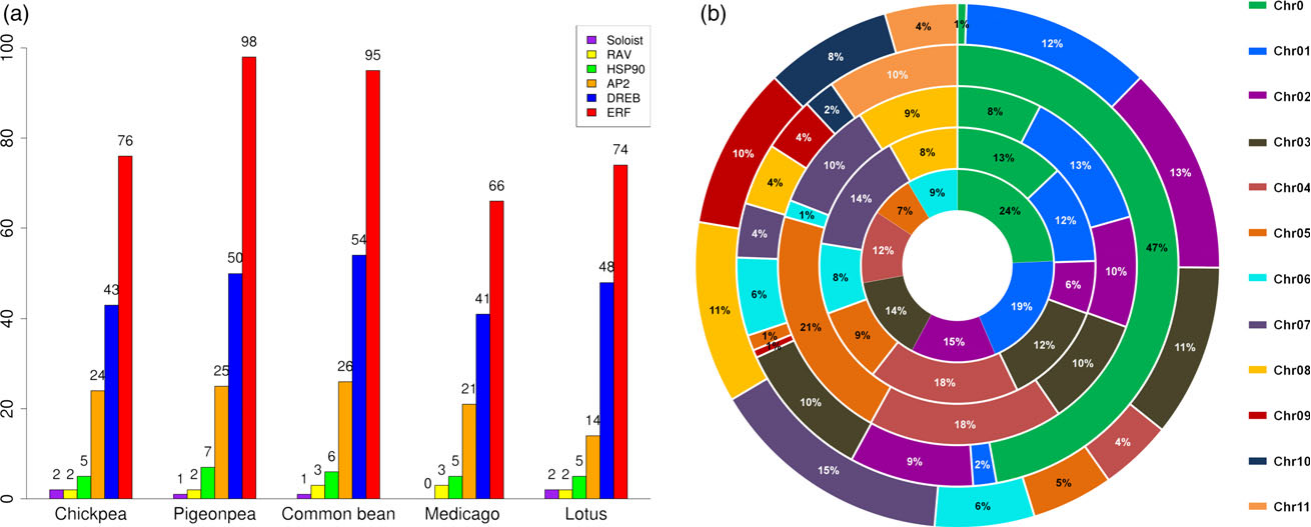
Distribution of genes encoding transcription factors of AP2/ERF family and HSP90 across five legumes. Graphical representation of number of AP2, ERF, DREB, RAV, soloist and HSP90 genes in chickpea, pigeonpea, common bean, Medicago and Lotus (a). Chromosomal distribution and percentage share of AP2/ERF genes in five different legumes. The innermost ring represent chromosomes of Lotus, followed by chickpea, Medicago, pigeonpea and the outer most represents common bean (b).

To identify AP2/ERF transcription factor (TF) superfamily genes, BLASTP and HMM searches were performed against reference genomes of chickpea, pigeonpea, Medicago, common bean and Lotus. To identify the members of AP2/ERF subfamilies, the sequences were checked for the presence of AP2 and B3 domains. Sequences with single AP2 domain were classified as ERFs and the ones with two AP2 domains were categorized as AP2, while sequences sharing AP2 and B3 domains were classified under RAV (related to ABI3/VP1) subfamily. Sequences having low homology with ERF members were termed as ‘soloist’. ERF proteins were further subclassified into ERF and DREB proteins based on the variation in the amino acid sequences. ERFs were most conspicuously distributed followed by DREBs and AP2s in genomes of legumes (Xu *et al*., 2011). Different AP2/ERF family members which include AP2, ERF, DREB and soloist were identified, and their chromosomal distribution in five legume crops was determined. As a result, a total of 147, 176, 131, 179 and 140 AP2/ERF family members were identified in chickpea, pigeonpea, Medicago, common bean and Lotus, respectively (Figure 1a; Table 1). The chromosomal distribution of this AP2/ERF family of TFs revealed their localization on the pseudomolecules and scaffolds (Figures 1b and S1–S5). In a separate study, 16 AP2 and 120 putative ERF TFs were identified in chickpea (Deokar *et al*., 2015). In that study, the AP2s were identified and characterized strictly based on the presence of two AP2 domains whereas in our present study, despite the presence of one AP2 domain, three ERFs in chickpea, two in pigeonpea and one in common bean were clustered with AP2 sequences. Similar observations were also made in the case of Arabidopsis, potato (*Solanum tuberosum*) and rubber (*Hevea brasiliensis*), where four, five and seven sequences with single AP2 domain, respectively, were classified as AP2 (Duan *et al*., 2013). One possible reason could be the presence of larger number of introns compared to other ERFs, which is a peculiar feature of AP2 sequences. In the case of soybean, 98 unigenes with full‐length AP2/ERF domains were identified in an earlier study (Zhang *et al*., 2008). Variations in biochemical attributes like isoelectric point, protein length and molecular weight of the members of same family indicate the presence of putative novel variants (Tables S1–S5) and are in accordance with the findings in foxtail millet (Lata *et al*., 2014).

**Table 1:** Summary of the structure of AP2/ERF transcription factor superfamily in five legumes

Subfamily	Subgroup	Chickpea	Pigeonpea	Common bean	Medicago	Lotus
DREB	A1	6	5	8	4	7
	A2	5	9	8	7	4
	A3	1	1	1	1	1
	A4	14	18	19	14	18
	A5	10	10	10	11	11
	A6	7	7	8	4	7
	Total	43	50	54	41	48
ERF	B1	12	16	17	17	12
	B2	5	5	4	6	4
	B3	23	39	33	16	26
	B4	14	8	9	6	7
	B5	8	7	8	5	6
	B6	14	23	24	16	19
	Total	119	148	149	107	122
AP2		14	16	16	14	11
AINTEGUMENTA		10	9	10	7	3
RAV		2	2	3	3	2
Soloist		2	1	1	0	2
Total AP2/ERF family genes		147	176	179	131	140
Total genes in genome		28 269	48 680	31 638	45 888	37 971
AP2/ERF transcription factor genes (%)		0.52	0.36	0.57	0.29	0.37
Genome size (Mb)		738	833	521	257.60	472
Average number of AP2/ERF TFs per Mb		0.20	0.21	0.34	0.51	0.30

We observed that the genome sizes and the number of gene family members of AP2/ERF were not directly correlated in these legumes. For instance, even though Medicago and chickpea have large variations in their genome size, did not show much variation in the number of AP2/ERF genes. Similarly, the number of AP2/ERF genes in common bean (521 Mb) did not differ significantly with the number of AP2/ERF genes in pigeonpea (833 Mb), although their genome sizes varied significantly. Nevertheless, in general, the cool season legumes (Lotus and Medicago) possessed low number of AP2/ERF members when compared to warm season legumes (pigeonpea and common bean). In spite of the considerable difference in genome size of respective legumes, little variation in the number of AP2/ERF transcription factors indicated that this family remained conserved during the evolution of legumes. RAV is considered to be one of the most conserved subfamilies among dicot species and is generally known to have six members (Licausi *et al*., 2010). The number of RAV genes (two to three) identified in this study was similar to what were found in dicots like tomato (*Solanum lycopersicum*) and potato. However, in Chinese cabbage (*Brassica pekinensis* L.) , as many as 14 RAV genes out of a total of 291 AP2/ERF genes were identified (Song *et al*., 2013). Additional information on the isoelectric points, molecular weight and variation in the amino acid sequences are provided as Supplementary information.

### Identification of HSP90 family genes

To identify HSP90 family in five legume species, protein sequences were scanned for the presence of histidine kinase‐like ATPases (HATPase_c) and HSP90 motifs. As a result, five HSP90 genes in chickpea, seven in case of pigeonpea, six in common bean, and five each in Medicago and Lotus were identified (Figure 1a). The proteins encoded by HSP90 genes ranged from 648 to 818 amino acids in length with isoelectric points ranging from 4.79 to 5.45 (Table S6), suggesting the conserved nature of HSP90 proteins across the five legumes. The number of amino acids in soybean HSP90s ranged from 699 to 847 (Xu *et al*., 2012). Interestingly, all HSP90 genes in common bean, Medicago and Lotus were found on the pseudomolecules, whereas three of the HSP90 genes identified in each chickpea and pigeonpea were found on pseudomolecules, while two and four HSP90 genes were identified on scaffolds, respectively (Figures S1–S5).

### Classification of ERF and DREB members

**Figure 2:**
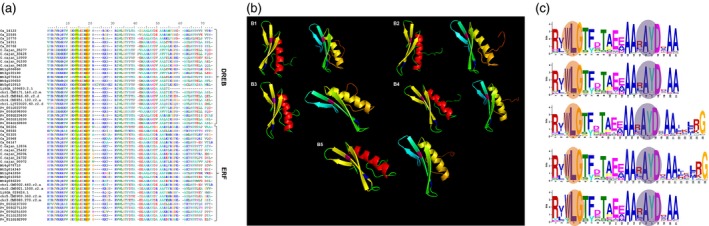
Sequence alignment, prediction of structure and conserved motifs of ERF and DREB proteins across five legumes. (a) Multiple sequence alignment (MSA) representative sequences of amino acid sequences of ERF and DREB subfamily proteins of chickpea, pigeonpea, Medicago, common bean and Lotus using ClustalW. (b) Conserved amino acids in ERF and DREB sequences across the five legumes chickpea (B1), pigeonpea (B2), Medicago (B3), common bean (B4) and Lotus (B5) predicted by I‐TASSER. Structures with red alpha helix represent DREB and yellow represents ERF. Pink and blue residues represent the conserved valine and glutamic acid on beta sheets of DREB; alanine and aspartic acid on beta sheets of ERF. (c) Highly conserved WLG and RAYD elements found in motif 1 of AP2/ERF domain across chickpea (C1), pigeonpea (C2), Medicago (C3), common bean (C4) and Lotus (C5).

In the present study, ERF and DREB members of ERF subfamily were distinguished based on the sequence alignment. The sequences with alanine and aspartic acid conserved at 14th and 19th position, respectively, were classified as ERF, while those with valine and glutamic acid conserved at 14th and 19th position were classified under DREB. In addition, the amino acids were also found to be conserved in the tertiary structure of these proteins (Figure 2a,b). The domains with conserved 14V, irrespective of a residue at 19th position were also classified as DREBs because of the importance of 14V over 19E in determining the DNA‐binding specificity of DREB transcription factor to the DRE *cis*‐element (Sakuma *et al*., 2002).

The conserved amino acids V14 and E19 in the ERF/AP2 domains of DREB proteins play a quintessential role in DNA binding and substitution at these amino acids with alanine (A) and aspartic acid (D), hallmark of ERF proteins leads to reduced DNA‐binding activity and specificity (Sakuma *et al*., 2002). Further, 16 conserved amino acids specific to ERF and DREB proteins were also identified in more than 90% proteins of the five legumes. Earlier studies in *Hevea brasiliensis* (Duan *et al*., 2013) identified ten such signature amino acids, 14 each in Arabidopsis, cotton (*Gossypium hisutum* L.) and rice (Champion *et al*., 2009) and were recognized as group markers of the ERF family. The sequence alignment also revealed two additional elements, WLG and RAYD which were conserved among the legumes, studied for most of the AP2/ERF family members (Figure 2c). More details are provided under the section on motif prediction.

### Phylogeny of AP2/ERF and HSP90 proteins

#### Phylogeny of AP2/ERF proteins

**Figure 3:**
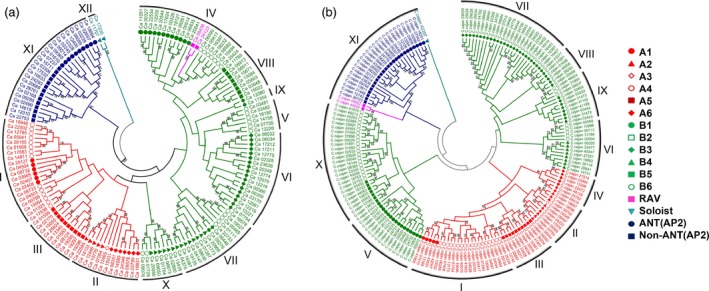
Phylogenetic analysis of AP2/ERF genes in chickpea and pigeonpea. Conserved domains of AP2/ERF genes were used to construct phylogenetic tree for chickpea (a) and pigeonpea (b). Legends on the right represent the respective subfamily members. Bootstrap values greater than 50% support are indicated.

Phylogenetic analysis based on conserved domains in DREB, ERF, AP2, and RAV subfamilies, grouped the AP2/ERF proteins of the five legumes into 11–15 groups. In the case of chickpea, AP2/ERF proteins were grouped into 12 major groups (Groups I–XII). Among these groups, Groups I–III comprised of DREB subfamily, Groups IV–X possessed both ERF and RAV subfamilies and Group XI consists of AP2 subfamily, while two soloists (Ca_11707 and Ca_17230) were placed in Group XII (Figure 3a). The AP2 family was further classified into two groups including ten AINTEGUMENTA (ANT) and 11 AP2 members. Three ERF sequences with single AP2 domain that clustered with AP2 sequences were considered as AP2s instead of ERFs. Eight members of the Group I were identified with a consensus core sequence ATDS [SD], a representative feature of cytokinin response factor (CRF) proteins (Liu *et al*., 2013; Table 2). To date, 21 BrCRFs (Liu *et al*., 2013), 12 AtCRFs and 11 SlCRFs (Shi *et al*., 2012) have been identified and characterized in detail. In general, the proportion of CRFs in AP2/ERF protein family is expected to be in the range of 5%–10%; for instance, rice and poplar (*Populus trichophora*) were reported to have 6.5% in each (Nakano *et al*., 2006; Zhuang *et al*., 2008). In our study, it was found to be 5.36% in chickpea. In case of chickpea, 37.5% CRFs were found to contain a C‐terminal SP[T/V]SVL motif, which functions as a putative MAP kinase and/or casein kinase 1 phosphorylation site speculated to be involved in cytokinin signalling pathway (Xu *et al*., 2008) along with CRF domain [ATDxSS] motif. In pigeonpea, all six CRFs were marked only by the presence of CRF domain, while the putative MAPK phosphorylation site was not present. In case of Medicago, common bean and Lotus the AP2/ERF sequences were not marked by the presence of either CRF or the putative MAPK phosphorylation domains. However, this doesn't rule out the possibility that ERFs without CRF domain will not respond to cytokinins, as reported in rice, where up‐regulation in AP2/ERF expression in response to cytokinins was reported despite the absence of CRF domains (Hirose *et al*., 2007).

**Table 2:** Summary of motifs identified specific to each subfamily in each legume crop

Subfamily	Subgroup	Motif number in legumes				
		Chickpea	Pigeonpea	Common bean	Medicago	Lotus
DREB	A1	7	8, 17	8, 10	7, 8, 15	7, 11, 15
	A2	14	14	13	–	–
	A3	–	–	–	–	–
	A4	7	8, 17	8, 10	7	7
	A5	7	8, 23	8	7, 23	7, 19
	A6	–	–	–	–	–
ERF	B1	–	–	–	13	12
	B2	–	–	–	–	–
	B3	13	9, 24	9	14, 17, 18, 19	17, 18
	B4	10	–	–	–	–
	B5	21	20	14	–	–
	B6	15	11, 16, 18	9, 4, 12, 15, 18, 20	8, 21	9, 13, 18, 20, 24
CRF		11	20	–	–	13
AP2		6, 8, 9	4, 5, 12, 15	4, 5, 6	4, 5, 9, 10, 11, 16	4, 6, 10, 14
AINTEGUMENTA		12	19	19	12	–
RAV		–	–	17	24	–

–, no motif identified.

DREBs were grouped as A1–A6 and ERFs were grouped as B1–B6 in all the legumes based on their phylogeny (Figure S6). DREBs (A1–A6) were classified mainly into Groups I to III in the case of chickpea, whereas these were grouped into Groups I to IV in the case of pigeonpea and Medicago and into Groups I to V in the case of common bean and Lotus. However, in case of peanut, two subgroups (A1 and A3) were found to be absent (Wan *et al*., 2014). A total of 11 major groups were identified (Groups I–XI) in pigeonpea. Groups I–IV consisted of DREBs, Groups V–X contained ERFs, Group XI contained AP2 and RAV, and one soloist remained ungrouped (C.cajan_42397). Two ERFs that clustered with AP2 group were considered as AP2. Eleven ANTs were found to cluster together in AP2 family. Six ERF members of Group I were identified with CRF domain (Figure 3b). Similar studies were also conducted in Medicago (Figure S7), common bean (Figure S8) and Lotus (Figure S9) for which details are provided as Supplementary information.

#### Phylogeny of HSP90 proteins

HSP90 proteins from all five legumes were analysed *in silico* for their location in cellular milieu using ProtComp v9.0 of Softberry (http://www.softberry.com/berry.phtml). In chickpea and pigeonpea, 3/5 and 4/7 proteins were predicted to be localized in cytoplasm, and the others were either localized on chloroplast (1/5 and 2/7) or endoplasmic reticulum (1/5 and 1/7). However, in Medicago, common bean and Lotus, 3/5, 3/6, and 2/5 proteins, respectively, were predicted to be cytoplasmically localized and the others on chloroplast, mitochondria and endoplasmic reticulum. None of the identified HSP90 proteins in chickpea and pigeonpea were predicted to be localized in mitochondria (Table S6). Based on subcellular organization, HSP90 proteins were grouped into two major groups (Groups I and II) using neighbour‐joining method. As shown in Figure S10a, Group I consisted of cytosolic and Group II consisted of organellar HSP90 proteins.

### Gene structure and motif prediction of AP2/ERF and HSP90 families

#### Gene structure of AP2/ERF

In chickpea, 53/79 ERFs were found to be intronless, and 23 had one intron. However, three ERF genes clustered with AP2 showed higher number of introns (seven to eight), as seen in AP2 sequences. 41/43 DREBs were intronless, and the rest two had one intron. In case of AP2 genes, introns ranged from 6 to 11 and none of them were intronless, while the two soloists contained three and five introns each (Table S1). In pigeonpea, 61/100 ERFs were intronless, 34 had one intron and three had two introns, and the two ERFs clustered with AP2 contained four and eight introns each. AP2 genes contained 5–12 introns, 38/50 DREB genes were intronless, 11 contained one intron each and the remaining one had two introns. Soloist contained only one intron (Table S2).

In Medicago, 31/67 ERF genes were without introns, 30 genes had one, and six genes contained two. None of the AP2 genes were intronless and introns ranged from 5 to 14, 31/41 DREBs were intronless, four had one intron and the other six genes had two introns (Table S3). In common bean, 68/94 ERFs were intronless, 21 ERFs contained one intron each, three had two and two had three introns. Forty‐seven of 55 DREB genes were intronless and the other eight had one intron. All 28 AP2 genes contained introns ranging from 6 to 18 (Table S4). The only soloist had 11 introns. Lotus had all 48 DREBs without introns, 58/74 ERFs were without introns, 15 had one intron and one gene was with two introns. All 17 AP2 genes contained two to nine introns (Table S5). The two soloists had one and five introns each. None of the RAV sequences in chickpea, Lotus and common bean contained introns. However, in pigeonpea, one to two introns were present in the identified two RAVs, and in Medicago, two of three RAVs were intronless, the other one contained only one intron.

In general, the ERF and DREB subfamily members outnumbered the AP2 and RAV subfamilies, which have more complex gene structure with two AP2 domains, more number of introns and a RAV‐specific B3 domain. The lesser number could be attributed to speculation of early addition of introns, or perhaps the second DNA‐binding structure resulting in impaired duplication of ancestral HNH endonuclease during early evolution of this family of genes. Otherwise, the transposition of longer DNA segment might have prevented the duplication, thus resulting in lesser number of AP2s and RAVs (Magnani *et al*., 2004).

#### Gene structure of HSP90

In the case of HSP90, the exon–intron boundaries of Group I consisted of lesser introns compared to Group II. Members of Group I had two to four introns, whereas Group II contained genes with 14–19 introns. Similarly, in soybean three such groups with genes having two to three introns in one Group, 14–16 in second Group and ≥18 in the third Group have been reported in an earlier study (Xu *et al*., 2012). The splicing phases were designated as: phase 0, splicing happened after third nucleotide of the codon; phase 1, splicing after first nucleotide of the codon; and phase 2, splicing after the second nucleotide. The splicing phases were conserved within Group I and Group II members, but showed stark differences among the other two groups (Figure S10b). The exon–intron organization of paralogous pairs of HSP90 present on pseudomolecules was also examined to identify traceable intron loss/gain within these genes. Intron loss/gain within one pair of paralogous genes was observed in chickpea (Ca_17680/Ca_09743). Two pairs of paralogous genes, each in pigeonpea (Cc_15978/Cc_07342, Cc_15978/Cc_05971) and common bean (Pv008G281300/Pv004G107700, Pv008G281400/Pv004G107700), showed intron loss/gain. One paralogous gene pair, each in pigeonpea (Cc_05971/Cc_07342), common bean (Pv008G281300/Pv008G281400) and Medicago (Mt5 g096430/Mt5 g096460), showed conserved exon/intron structures in terms of number of introns. Interestingly, Mt1 g099840 contained an additional C‐terminal exon, in comparison with other Group I members. No paralogous genes/duplications events were seen in Lotus (Table S7).

#### Motif prediction

A total of 25 motifs were screened for each legume using MEME (default parameters). Among them, two motifs (motif 1 and 2) were seen in almost all AP2/ERF members in chickpea, pigeonpea, Medicago, common bean and Lotus. A total of 146/147, 176/176, 120/131, 179/179 and 135/140 AP2/ERF members in chickpea, pigeonpea, Medicago, common bean and Lotus, respectively, contained motif 1 (Figures S11–S15). The other motif was observed in 137/147, 169/176, 177/179, 131/131 and 119/140, genes in respective legumes as specified above. Some WLG elements (motif 1) (Figure 2c) were found to be converted into YLG elements in AP2 subfamily. Further, the conserved RAYD (motif 1) element in AP2/ERF superfamily was converted to RAHD in a very few sequences, contrary to complete conversion to RAHD in two subgroups of DREB in Chinese cabbage (Song *et al*., 2013). Motifs 5, 6, 8 and 9 were chickpea AP2 subfamily‐specific, shared by 22, 18, 16 and 8 members, respectively. Similarly, motifs 4, 5, 12 and 15 were present in 18, 20, 5, 11 and 22 pigeonpea AP2 sequences; in Medicago, motifs 4 and 5 were shared by 16 and 12 members; common bean shared 4th and 6th motif by 17 and 18 proteins of this subfamily; and in Lotus, motif 4 and 6 were shared by 13 and 6 AP2 proteins. Of 25 motifs predicted, 7th motif was DREB‐specific in chickpea and observed in 27 proteins and was also shared by 25 members in Lotus. Motif 8 was shared by 26, 29 and 21 DREB members in pigeonpea, common bean and Medicago, respectively. More elaborative motif distribution in each of the five legumes is listed in Table 2. CRF‐specific N‐terminus [ATDxSS] domain was found in four legumes except Medicago. However, the TEH motif at the start of N‐terminus was missing, which is in accordance with earlier findings (Rashotte and Goertzen, 2010; Zwack *et al*., 2012). The ERF associated amphiphilic repression (EAR) motif known to repress the transcription (Ohta *et al*., 2001), like DEAR1, a DREB sequence containing EAR motif mediates crosstalk between signalling pathways for stress responses (Tsutsui *et al*., 2009). Similar results have been reported in rubber (Duan *et al*., 2013), tomato (Sharma *et al*., 2010) and Arabidopsis (Licausi *et al*., 2010).

The RAV subfamily is known to regulate gene expression in response to ethylene (Alonso *et al*., 2003), other biotic (Sohn *et al*., 2006) and abiotic stresses (Li *et al*., 2011), by binding to a bipartite recognition sequence with the B3 and AP2 recognizing the sequences, CACCTG and CAACA. The motif [YEAHLWD] specific to AP2 subfamily in chickpea, pigeonpea, Medicago, common bean and Lotus are known to form a long linker between the two β‐sheets like the linker residues in AINTEGUMENTA (ANT) protein, expected to be involved in activating the function of TFs in AP2 subfamily. Another motif, [IHEYQAKS] LNFP was found specific to ERF subfamily among the five concerned legumes. The motif is found to be characterized by three blocks of conserved amino acid residues: LPRP, D [IV] QAA/DIR [RA] specific to ERF and [IHEYQAKS] LNFP specific to DREB. These residues are known to interact with CBL‐interacting serine/threonine proteins kinase‐12 (Albrecht *et al*., 2001) and ethylene‐responsive factor, ERF037 (Qu and Zhu, 2006). Xu *et al*. (2013) also identified similar motifs in castor bean (*Ricinus communis* L.). Details of motif prediction in case of HSP90 genes are provided in Supplementary information. Gene ontology analysis indicated that large number of genes were annotated for biological processes in all five legumes (Figure S16; Tables S18–S22).

### Chromosomal distribution, duplication and orthologs of AP2/ERF and HSP90 genes

Of 147 AP2/ERF and five HSP90 genes identified in chickpea, 128 and three were found on eight chickpea pseudomolecules (Ca1–Ca8). Similarly, 93/176, 3/7 in pigeonpea, 122/13, 4/6 in Medicago, 179/179, 6/6 in common bean and 105/140, 5/5 in Lotus, AP2/ERF and HSP90 genes could be found on the pseudomolecules. In chickpea, maximum number (27) of genes with 16 ERFs, eight DREBs and one each of RAV, AP2 and HSP90 were located on Ca4, followed by Ca7 (21 genes) and Ca3 (18 genes). Other two HSP90s were identified on Ca2 and Ca5. Six AP2/ERF genes including three ERFs, two DREBs and one AP2 (Ca_09050, Ca_09076, Ca_09124, Ca_09214, Ca_14911 and Ca_18387) were identified in the ‘*QTL‐hotspot*’ region (35.8–46.7 Mb) on chromosome 4 (Table S1) of chickpea for drought tolerance (Kale *et al*., 2015). Chromosomal distribution of genes in pigeonpea was maximum on CcLG2 and CcLG3, with 18 genes each including, six ERFs, seven DREBs, two each of AP2s and HSP90s and one RAV on CcLG2 and 11 ERFs, four AP2s and three DREBs on CcLG3, followed by 17 genes on CcLG11. The other HSP90 gene was found on CcLG8. Gene distribution in Medicago was found to be maximum on Mt5 (*Medicago truncatula* chromosome 5) with 31 including, eight ERFs, 16 DREBs, three AP2s, one RAV and three HSP90 genes followed by Mt4 (23 genes) and Mt1 (19 genes including one HSP90 genes). In case of common bean, maximum number of genes (27) were identified on Pv7 (*Phaseolus vulgaris* chromosome 7) with 19 ERFs, five DREBs, two RAVs and one AP2 followed by Pv2 and Pv8 (24 and 23 respectively). The HSP90 genes were found on Pv1, Pv2 and Pv4. Lotus shared the maximum number of genes (27) on Lj1 (*Lotus japonicus* chromosome 1) with 18 ERFs, nine DREBs, four AP2s, and one soloist followed by Lj2 (24 including four HSP90 genes) and Lj3 with 20 genes.

**Figure 4:**
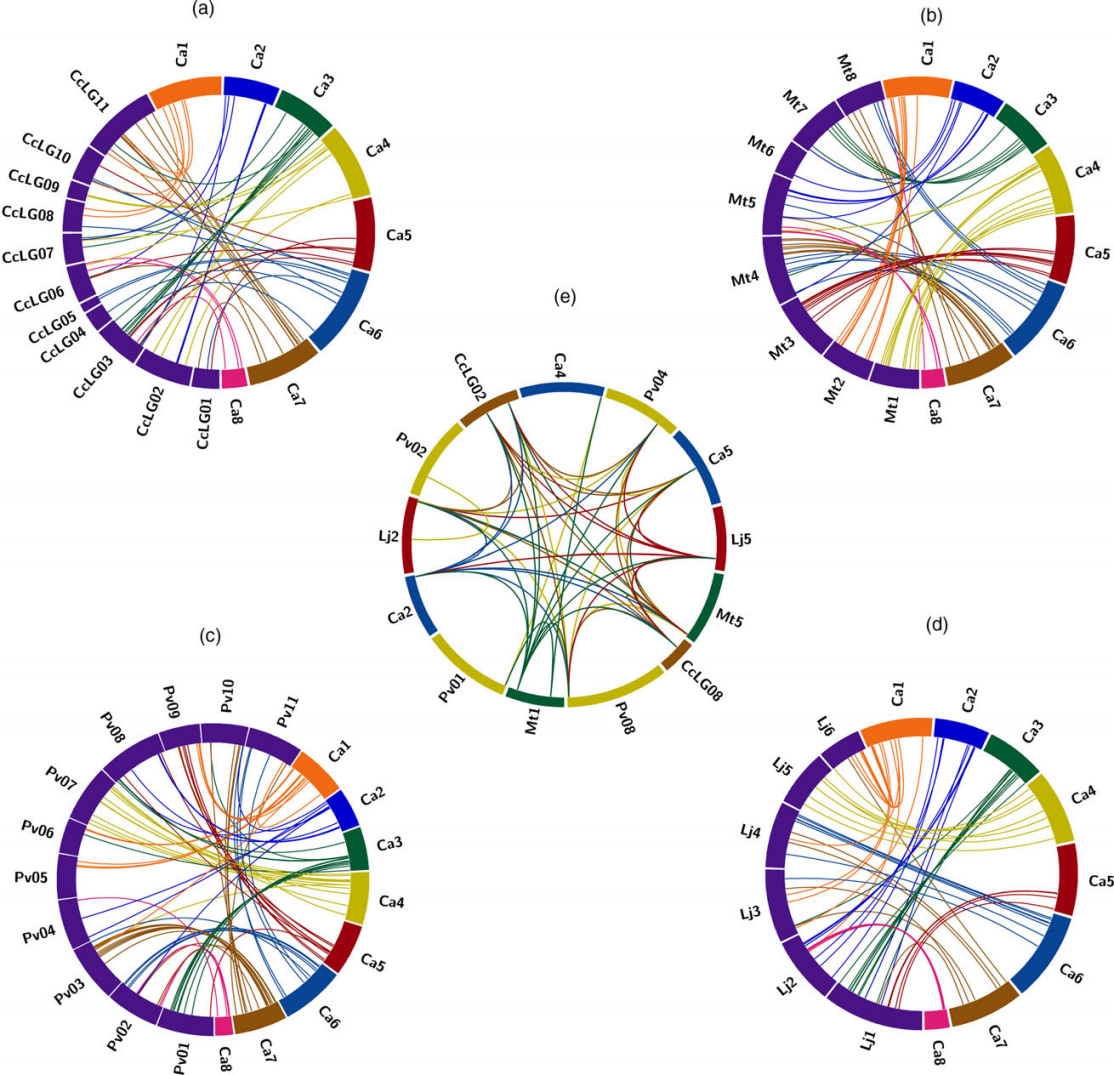
Comparative analysis of orthologous relationship among five legumes for AP2/ERF and HSP90 genes. The Circos plot represents orthology of chickpea genes with (a) pigeonpea, (b) Medicago, (c) common bean and (d) Lotus. Eight pseudomolecules of chickpea are represented with different colour and chromosomes of four species in blue. The strokes originating with the same colour from chickpea pseudomolecules landing on a different species represent an orthology of a given gene between the two species. (e) Orthologous relationships among the five legumes for HSP90 genes.

To identify the contribution of segmental and tandem gene duplications in genome‐wide expansion of AP2/ERF family in the considered five legumes, genes which were found within the 5‐Mb regions with 80% and higher similarity with e‐value threshold of 1e‐10 were considered as tandemly duplicated genes, and the ones separated by >5 Mb distance were identified as segmentally duplicated genes (Figures S1–S5). We found a total of 13 duplication events (paralogous genes) in chickpea, 18 in pigeonpea, 14 in Medicago, 13 in common bean and 17 in Lotus (Table S7). Of these duplication events, two groups of tandemly duplicated ERF genes in chickpea, one ERF in pigeonpea, three in Medicago (two of ERF and one of HSP90), one group of HSP90 genes in common bean and two in Lotus, and one group each of ERF and DREB genes were identified. It was observed that most of the groups were formed by ERF subfamily. However, in case of Medicago and common bean, one group of HSP90 genes was also identified, apart from just one tandemly duplicated group of DREB genes identified in Lotus among the five legumes (Figures S1–S5). HSP90 genes were segmentally duplicated compared to tandem duplications. It is obvious from these findings that segmental duplications outnumbered the tandem duplications, thus signifying a major role of segmental duplications in expansion of this gene family. Orthologs of chickpea in pigeonpea, Medicago, common bean, and Lotus were found using the best bidirectional BLAST approach with an e‐value threshold of 1e‐10. We identified, 100, 103, 84 and 80 such orthologs of chickpea AP2/ERF in pigeonpea, Medicago, common bean, and Lotus (Figure 4a–d; Table S8). Similarly, six orthologs of chickpea HSP90 were found in Medicago, pigeonpea and common bean and three in Lotus (Figure 4e; Table S9). All the legumes considered in this study were identified with segmental duplications to play a key role in the expansion of AP2/ERF family. Similar results have been reported in rice (Sharoni *et al*., 2011), Arabidopsis (Nakano *et al*., 2006) and *Brassica rapa* ssp. pekinensis (Liu *et al*., 2013), indicating that mechanisms underlying AP2/ERF family expansion vary from species to species.

### Gene expression patterns in chickpea

**Figure 5:**
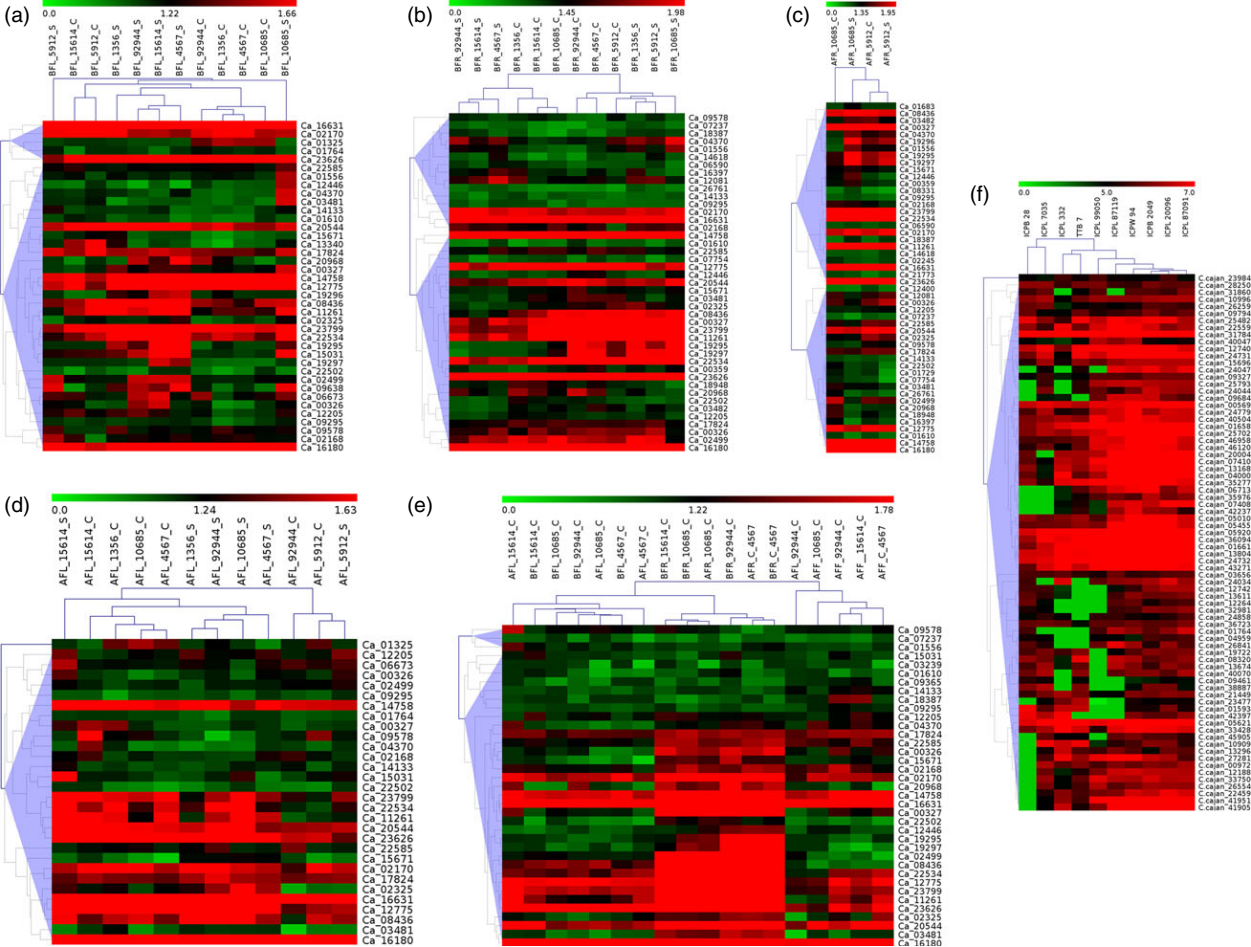
Expression profiles of AP2/ERF and HSP90 genes in chickpea and pigeonpea. Hierarchical clustering of AP2/ERF and HSP90 genes in chickpea (a–e) and pigeonpea (f) using log_10_‐transformed FPKM values. BFL, before flowering in leaf; BFR, before flowering in roots; AFL, after flowering in leaf; AFR, after flowering in roots; C, control; S, stressed. (a) Two genes, namely Ca_09638 and Ca_02499, were found to be specifically expressed in the stressed leaf tissues, while repressed in the control. (b) In root tissues, Ca_04370 was found to be specifically expressed under stress. (c) Three genes, Ca_19295, Ca_19296 and Ca_19297, were found to be highly expressed in all the stressed root tissues. (d) Ca_02325 showed specific expression, while Ca_08436 was highly expressed in comparison with control leaf tissues. (e) A gene cluster was identified which is specifically expressed in root tissues unlike genes Ca_16180, Ca_16631 and Ca_14758, which were constitutively expressed in all the tissues under controlled conditions irrespective of the developmental stages. (f) C.cajan_24047 showed high expression in all the Fusarium wilt‐resistant genotypes, while repressed in the susceptible ones. Two genes, C.cajan_25793 and C.cajan_24044, were highly expressed in the SMD‐resistant genotypes when compared to the susceptible genotypes.

To gain insights into the expression pattern of AP2/ERF and HSP90 genes in chickpea under heat stress, RNA‐seq data generated from leaf, root and flower tissues at vegetative and reproductive stages from three tolerant (ICCV 92944, ICC 1356, ICC 15614) and three sensitive (ICC 5912, ICC 4567, ICC 10685) genotypes was used. Expression patterns were compared between respective controls and (i) heat‐stressed leaf tissue before flowering, (ii) heat‐stressed root tissue before flowering, (iii) heat‐stressed root tissue after flowering, (iv) heat‐stressed leaf tissue after flowering and (v) vegetative (leaf and root) and reproductive (flower) tissues in two heat‐tolerant and one heat‐sensitive chickpea genotype (Figure 5a–e; Tables S10–S14). Unique set of 58/147 AP2/ERF genes were expressed in different tissues in chickpea with 39, 43, 50, 30 and 37 genes in each of the five comparisons as mentioned above. Hierarchical clustering for each comparison broadly classified them into clusters representing gene expression levels.

**Figure 6:**
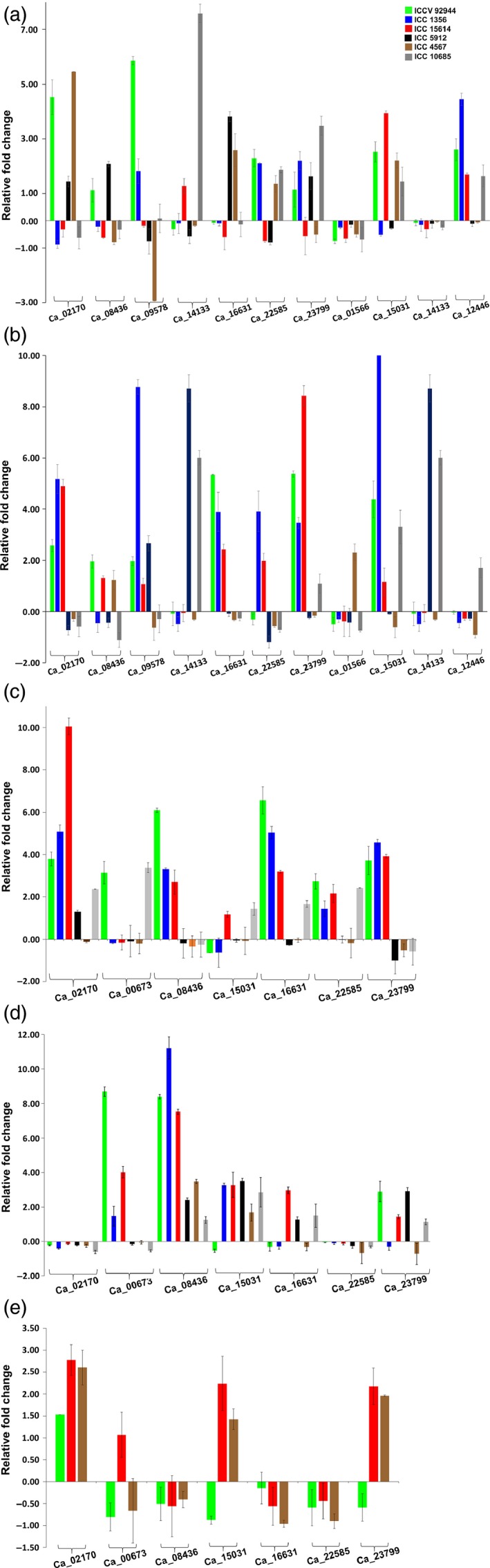
Expression profiling of AP2/ERF genes in chickpea. Quantitative real‐time PCR validation of differential expression of genes in vegetative leaf (a), vegetative root (b), reproductive leaf (c), reproductive root (d) and flower (e) tissues in heat‐ tolerant and ‐sensitive chickpea varieties. Expression in flower tissues was performed in two tolerant and one sensitive genotype. The other genotypes could not withstand the heat stress till flowering stage.

Validation using qRT‐PCR (Primers sequence provided in Table S15) demonstrated differential, temporal, spatial and genotype‐specific expression of genes (Figure 6). In vegetative leaf tissue, Ca_01566 and Ca_14133 were down‐regulated among all the six heat‐tolerant and heat‐sensitive genotypes, whereas Ca_14133 was significantly up‐regulated in one of the three sensitive genotypes, and Ca_02170 was up‐regulated in one tolerant and two sensitive genotypes (Figure 6a). In vegetative root tissue, Ca_02170 and Ca_16631 were up‐regulated in tolerant and almost negligible expression in sensitive genotypes when compared to its control. However, Ca_09578 and Ca_14133 were up‐regulated in all tolerant and one sensitive genotype and significantly up‐regulated in two sensitive genotypes, respectively, while Ca_22585 and Ca_23799, on the other hand, were down‐ and up‐regulated in sensitive and tolerant genotypes, respectively (Figure 6b). Ca_15031 was found to be significantly up‐regulated in one tolerant genotype with almost more than 10‐fold expression (Figure S6c). In case of reproductive leaf tissue, Ca_02170 was up‐regulated in both tolerant and sensitive genotypes except one sensitive genotype where the expression was almost negligible, and Ca_08436 and Ca_23799 were up‐regulated in tolerant and down‐regulated in sensitive genotypes (Figure 6d). In flower tissues, Ca_02170 was almost unexpressed and Ca_00673 was up‐regulated in tolerant and with almost zero expression in sensitive genotypes, whereas Ca_08436 and Ca_15031 were up‐regulated and Ca_22585 was insignificantly down‐regulated across all genotypes (Figure 6e). Expression results prompt the identification of probable tissue and stage‐specific candidate genes which can counteract the given stress condition. Similar studies in peanut against heat stress resulted in stress tolerant *AhERF019* transgenic Arabidopsis plants (Wan *et al*., 2014). In soybean, expression analysis and transgenic tobacco plants developed using GmERF057 and GmERF089 revealed enhanced tolerance to salt and drought stress, but not to pathogen stress under GmERF089 overexpression. However, GmERF057 overexpression resulted in enhanced tolerance to salt and pathogen stress (Zhang *et al*., 2008), conferring different roles of ERFs under different stress conditions. Another study in soybean showed transactivation of DREB2A;2 under drought, heat and low temperature (Mizoi *et al*., 2013).

**Figure 7:**
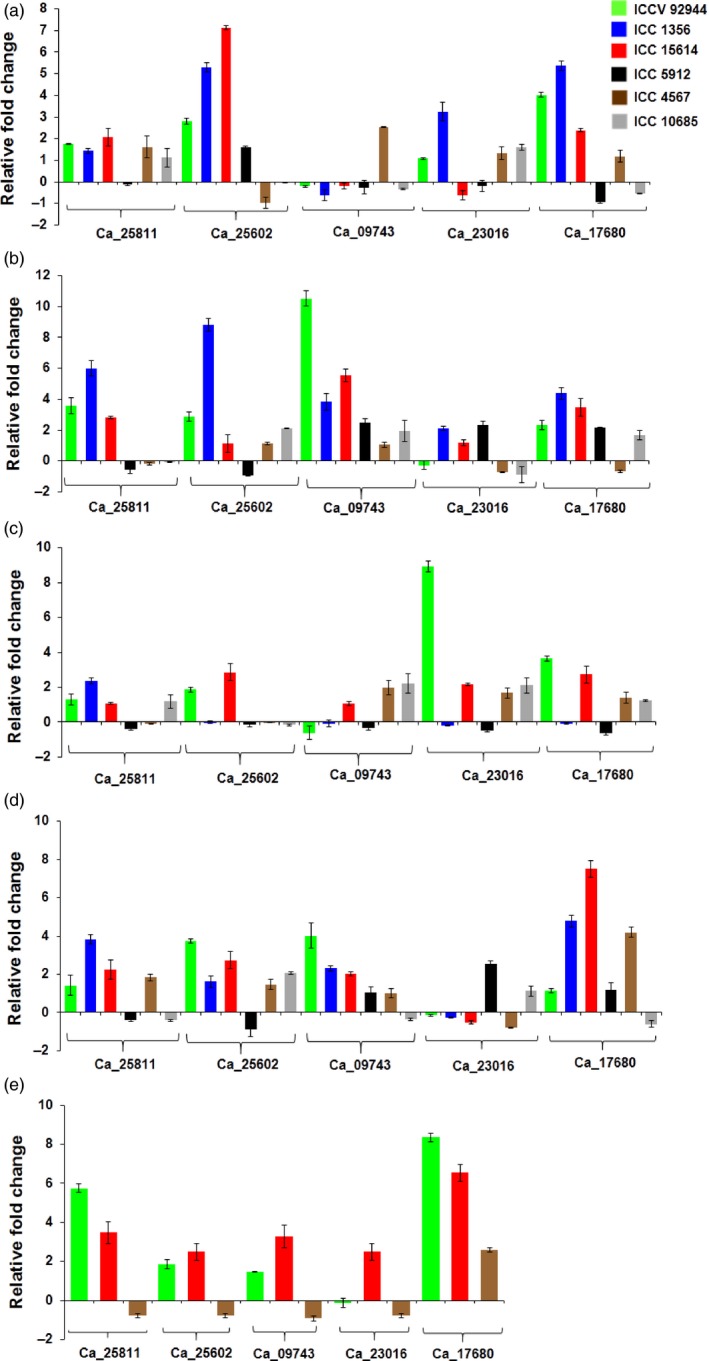
Expression profiling of HSP90 genes in chickpea. Quantitative real‐time PCR validation of differential expression of genes in vegetative leaf (a), vegetative root (b), reproductive leaf (c), reproductive root (d) and flower (e) tissues in heat‐tolerant and heat‐sensitive chickpea varieties. Expression in flower tissues was performed in two tolerant and one sensitive genotype. The other genotypes could not withstand the heat stress till flowering stage.

HSP90 genes in flower tissues of heat‐ tolerant and ‐sensitive chickpea genotypes were observed to be up‐regulated in tolerant compared to the sensitive genotypes, except Ca_17680 (Figure 7). Its expression was found to be induced even in the sensitive genotype compared to its control. However, expression of the same HSP90 genes in the vegetative leaf and root tissues were found to be up‐regulated, compared to control except for Ca_09743 (Figure 7a,b). It was observed to be up‐regulated in root and down‐regulated in leaf tissue. In reproductive leaf and root tissues, Ca_25602 and Ca_23016 were found to be consistently almost negligibly expressed or down‐regulated across the tolerant and sensitive genotypes (Figure 7c,d). Ca_25602 was up‐regulated in the two tolerant genotypes, whereas Ca_23016 was up‐regulated in a sensitive genotype (Figure 7e). Overall, the HSP90 genes were found to be up‐regulated across all genotypes and tissues. We observed that there was no single gene which was consistently up‐ or down‐regulated throughout the tissues and genotypes suggesting that HSP90 genes have temporal, spatial and genotype‐specific gene expression in chickpea. Ca_25602 in vegetative leaf, Ca_09743 in vegetative root, Ca_17680 in flower, Ca_23016 in reproductive leaf and Ca_17680 in reproductive root were found to be up‐regulated in heat‐stressed tissues compared to their control.

### Gene expression patterns in pigeonpea

A total of 76 AP2/ERF genes were quantified and depicted through heatmaps based on their FPKM values in ten pathogen‐stressed pigeonpea genotypes (Figure 5f; Table S16), which included five parental combinations (see materials and methods). Hierarchical clustering formed clusters of genes based on their FPKM values. Among the contrasting parents, ICPL 20096 and ICPL 332 showed the most contrasting expression followed by ICPB 2049 and ICPL 99050.

**Figure 8:**
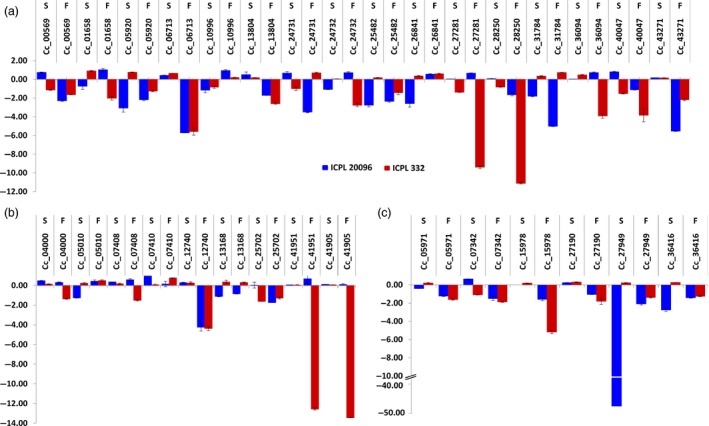
Expression profiling of AP2/ERF and HSP90 genes in pigeonpea. Quantitative real‐time PCR validation of differential expression of ERF genes (a), DREB genes (b) and HSP90 genes (c). F and S denote FW and SMD.

Among the resistant and susceptible genotypes, genes were mostly found to be up‐regulated in susceptible ones (Table S16). Validation using qRT‐PCR of 16 ERF, nine DREB and six HSP90 genes in ICPL 20096 and ICPL 332 was performed (Primers sequence provided in Table S17; Figure 8a–c). FW and SMD stress imposed root and leaf tissues from the two genotypes were analysed for the expression analysis. HSP90s also showed their role in disease resistance in several plant species. High throughput virus‐induced gene silencing in plants is implicated through HSP90 in disease resistance, like in case of barley for powdery mildew resistance (Hein *et al*., 2005; Lu *et al*., 2003). Similarly, in wheat, cytosolic HSP90 genes are known to be involved in seedling growth and disease resistance (Wang *et al*., 2011). The TF and chaperone genes in general, were observed to be down‐regulated in both the genotypes under biotic stress conditions instead of an expected up‐regulation. Five genes (C.cajan_06713, C.cajan_27281, C.cajan_28250, C.cajan_36094 and C.cajan_25702) in particular, showed a profound dip in expression in the FW‐stressed leaf tissues of ICPL 22096 genotype. Of these DNA‐binding TF genes, the one with the maximum down‐regulation (C.cajan_36094) is known to negatively regulate the transcription, and ethylene‐mediated signalling is also known to play role in defence response during respiratory burst and induced systemic resistance. Interestingly, the gene was slightly up‐regulated in leaf tissues in both the genotypes against viral infection, however, was down‐regulated in root tissues against fungal invasion with deep repression in ICPL 20096 and a minor repression in ICPL 332. Meanwhile, C.cajan_27281 was found to be significantly down‐regulated in root tissues in the resistant genotype (ICPL 22096) but was found to be slightly up‐regulated in the susceptible genotype (ICPL 332). An obvious trend of down‐regulation of AP2/ERF and HSP90 genes in the stressed tissues of these genotypes prompt towards a more complex mechanism of resistance and susceptibility against biotic stresses like FW and SMD mediated by this class of TF and chaperones in pigeonpea. Another gene (C.cajan_27949) with 50‐fold down‐regulation observed in ICPL 22096 is a HSP90 gene which is known to interact with a NBS‐LRR protein, RPM1 in Arabidopsis and a mis‐sense mutation in HSP90 resulted in diminished levels of RPM1 (Hubert *et al*., 2003).

## Experimental procedures

### Identification of AP2/ERF and HSP90 proteins from five legume proteomes

Two different approaches were used to mine the AP2/ERF and HSP90 domain containing sequences in chickpea, pigeonpea, common bean, Medicago and Lotus. (i) BLASTP search (e‐value 1e‐5) using Arabidopsis and rice AP2/ERF and HSP90 sequences. (ii) Hidden Markov model (HMM) scan using AP2 (PF00847) and HSP90 (PF00183) Pfam profiles. A final unique set of the protein sequences identified using above approaches were further scanned for the presence of AP2/ERF, HSP90 and HATPase_c domains. Only the sequences containing these domains were retained.

### Classification of AP2/ERF and HSP90 genes

Phylogenetic and evolutionary analyses were carried out by using MEGA6.0 (http://www.megasoftware.net/). Neighbour‐joining method with pairwise deletion option was used for construction of phylogenetic trees for all five legumes using domain peptide sequences of AP2/ERF. Reliability of the constructed trees was assessed by using boot strapping with 5000 replicates. The conserved motifs of AP2/ERF and HSP90 were predicted using standalone version of motif based sequence analysis tool (MEME) (version 4.9.0) (Bailey *et al*., 2009) with default parameters, number of motifs set at 25, optimum width of 10–200 amino acids and any number of repetitions of a motif. Gene structure prediction was made using online server, Gene Structure Display Server based on full‐length mRNA alignments with corresponding genomic sequences. Protein structures of AP2/ERF were predicted using I‐TASSER (Zhang, 2008) and viewed using PyMOL version 1.5.0.4 (www.pymol.org).

### Identification of orthologous AP2/ERF and HSP90 genes, their distribution and duplication

Generic feature format (GFF) files for the genomes of five legumes were used to mark the location of each gene on their physical maps. The distribution of AP2/ERF and HSP90 genes was visualized using MapChart (Voorrips, 2002). Orthologous genes with respect to chickpea were predicted using best bidirectional hit (BBH) approach with e‐value threshold of 1e‐10. The chickpea AP2/ERF genes were used as query against the database of pigeonpea, Medicago, common bean and Lotus AP2/ERF genes. The predicted orthologous genes were depicted using Circos program (Krzywinski *et al*., 2009). The AP2/ERF and HSP90 genes in each legume species were searched for duplication events at an e‐value of ≤1e‐10 and sequence identity of ≥80%.

### Gene expression studies

Gene expression patterns of AP2/ERF and HSP90 genes in chickpea were studied using RNA‐seq data generated from leaf, root and flower tissues at vegetative and reproductive stages, while in case of pigeonpea RNA‐seq data downloaded from sequence read archive (http://www.ncbi.nlm.nig.gov/sra) (SRA030523.1 to SRP005971.1) were used. RNA was isolated in three biological replicates from each sample. The quality filtered reads were mapped to respective chickpea and pigeonpea genomes with spliced read mapper, TopHat (Trapnell *et al*., 2009). Cufflinks followed by cuffcompare (Trapnell *et al*., 2010) was used to estimate the abundance of reads mapped to genes by calculating FPKM values (fragments per kilobase of transcript per million). Genes with class code ‘=’ were considered for expression studies. The heatmaps showing expression profiles were generated by using log_10_‐transformed FPKM values by MultiExperiment Viewer (MeV 4.8.1; Saeed *et al*., 2003).

### Validation of gene expression profiles using qRT‐PCR

Six chickpea genotypes, three tolerant (ICCV 92944, ICC 1356, ICC 15614) and three sensitive (ICC 5912, ICC 4567, ICC 10685) to heat as mentioned above, were used to validate the expression profiles of select candidate genes. Plants of each genotype were grown in five replications each for vegetative and reproductive stages. Three seeds of each genotype were sown in a pot (2.4 L volume) containing a mixture of black Vertisol soil, sand and vermicompost (4 : 2 : 1 by volume) and the plants were grown at 27/16 °C in a greenhouse for 20 days and then transferred to a growth room to expose them to high temperatures at vegetative stage. The control plants were continued to grow in the glasshouse at 27/16 °C. The temperature in the growth room was increased daily by 1 °C, for example 28–40 °C during the day and 16–25 °C during night. Therefore, the plants were exposed to a gradual increase in temperature for stress imposition. Leaf and root tissues at vegetative stage were harvested 15 days after heat stress imposition. Similarly, for reproductive stage, the plants were grown in greenhouse conditions until the first appearance of flowers. Then the plants were subjected to heat stress in growth room for 15 days as described above. Leaf, root and flower tissues were harvested at reproductive stage. At least three biological replicates of each tissue sample were harvested and stored at −80 °C until RNA extraction. In case of pigeonpea, two genotypes, one resistant and one susceptible, namely ICPL 20096 and ICPL 332, respectively, were used to validate the putative candidate genes identified in the present study. FW and SMD stresses were imposed on 10‐day‐old seedlings of ICPL 20096 and ICPL 332 grown separately for each stress. Root dip inoculation (FW) and leaf staple techniques (SMD) were followed for stress imposition under glasshouse conditions, and tissues (roots—FW; and leaves—SMD) were harvested after 7 days of stress. The seedlings were stressed with *Fusarium udum* Butler (6 × 10^6^ conidia/ml) for FW inoculation (Sharma *et al*., 2012) and with viruliferous mites (*Aceria cajani*) for SMD infection, at the two‐leaf stage by stapling the primary leaves with SMD‐infected pigeonpea leaves containing at least five live mites (Nene and Reddy, 1976). The RNA‐seq data for the selected genes were validated through qRT‐PCR using Applied Biosystems 7500 Real‐Time PCR System with the SYBR green chemistry (Applied Biosystems, Foster City, CA). The gene‐specific primers were designed using Primer3 software (http://bioinfo.ut.ee/primer3-0.4.0/primer3/) applying the default parameters with slight modification which includes product size 80–150 bp and primer size 18–25 bp (Tables S21 and S22). The qRT‐PCRs were performed using SYBR Green Master Mix in 96‐well plates with two technical replicates and three biological replicates using GAPDH (chickpea) and actin (pigeonpea) as endogenous controls. The PCR conditions used are as follows: 2 min at 50 °C, 10 min at 95 °C, and 40 cycles of 15 s at 95 °C and 1 min at 60 °C. The relative transcriptional level in terms of fold change was calculated using the $2^{-\Delta \Delta C_{T}}$ method (Livak and Schmittgen, 2001).

## Conflict of interest

The authors declare that they have no competing interests.

## Supporting information


**Figure S1** Genome‐wide distribution of AP2/ERF and HSP90 genes in chickpea. A total of 128 AP2/ERF and three HSP90 genes were found to be anchored onto the pseudomolecules, while the remaining (19 AP2/ERF and two HSP90) genes were localized on the scaffolds. Clusters of tandemly duplicated genes are highlighted in green and those linked with lines represent segmentally duplicated genes.

**Figure S2** Genome‐wide distribution of AP2/ERF and HSP90 genes in pigeonpea. A total of 93 AP2/ERF and three HSP90 genes were found to be anchored onto the pseudomolecules, while the remaining (83 AP2/ERF and four HSP90) genes were localized on the scaffolds. Clusters of tandemly duplicated genes are highlighted in green and those linked with lines represent segmentally duplicated genes.

**Figure S3** Genome‐wide distribution of AP2/ERF and HSP90 genes in Medicago.

**Figure S4** Genome‐wide distribution of AP2/ERF and HSP90 genes in common bean.

**Figure S5** Genome‐wide distribution of AP2/ERF and HSP90 genes in Lotus.

**Figure S6** Pie chart representation of DREB (A1–A6) and ERF (B1–B6) genes in five legumes.

**Figure S7** Phylogenetic tree based on conserved domain sequence of AP2/ERF protein in Medicago. The unrooted tree was divided into 12 groups, ERF (marked in green), DREB (marked in red), AP2 (marked in blue), RAV (marked in pink) and soloist (marked in teak). Legends on the right represent the respective subfamily members. Only bootstrap values greater than 50% support are indicated.

**Figure S8** Phylogenetic tree based on conserved domain sequence of AP2/ERF protein in common bean. The unrooted tree was divided into 12 groups, ERF (marked in green), DREB (marked in red), AP2 (marked in blue), RAV (marked in pink) and soloist (marked in teak). Legends on the right represent the respective subfamily members. Only bootstrap values greater than 50% support are indicated.

**Figure S9** Phylogenetic tree based on conserved domain sequence of AP2/ERF protein in Lotus. The unrooted tree was divided into 12 groups, ERF (marked in green), DREB (marked in red), AP2 (marked in blue), RAV (marked in pink) and soloist (marked in teak). Legends on the right represent the respective subfamily members. Only bootstrap values greater than 50% support are indicated.

**Figure S10** Phylogenetic relationships, gene structures and motif composition of HSP90 genes in chickpea (Ca), pigeonpea (Cc), common bean (Pv), Medicago (Mt) and Lotus (Lj). (a) Phylogenetic tree constructed using MEGA 5.0 by neighbour‐joining (NJ) method with 1000 bootstrap replicates. Bootstrap support is indicated at each node. (b) Exon/intron structures of the HSP90 genes. Blue boxes represent exons and black lines represent introns. The numbers indicate the splicing phases of HSP90 genes: 0, phase 0; 1, phase 1; and 2, phase 2. (c) Schematic representation of conserved motifs (obtained using MEME) in HSP90 proteins. Different motifs are represented by boxes of different colours.

**Figure S11** Putative motif prediction in chickpea using MEME.

**Figure S12** Putative motif prediction in pigeonpea using MEME.

**Figure S13** Putative motif prediction in Medicago using MEME.

**Figure S14** Putative motif prediction in common bean using MEME.

**Figure S15** Putative motif prediction in Lotus using MEME.

**Figure S16** Gene ontology assignment to the AP2/ERF sequences identified in the five legumes.

**Table S1** Physio‐chemical and structural properties of the identified AP2/ERF members in chickpea.
**Table S2** Physio‐chemical and structural properties of the identified AP2/ERF members in pigeonpea.
**Table S3** Physio‐chemical and structural properties of the identified AP2/ERF members in Medicago.
**Table S4** Physio‐chemical and structural properties of the identified AP2/ERF members in common bean.
**Table S5** Physio‐chemical and structural properties of the identified AP2/ERF members in Lotus.
**Table S6** Physio‐chemical properties of the identified HSP90 members in chickpea, pigeonpea, Medicago, common bean and Lotus.
**Table S7** List of AP2/ERF and HSP90 genes paralogs in chickpea, pigeonpea, common bean, Medicago and Lotus.
**Table S8** List of chickpea AP2/ERF orthologs in other legumes.
**Table S9** List of HSP90 orthologs among the five legumes.
**Table S10** Log_10_‐transformed FPKM values of control and heat‐stressed before flowering leaf tissues (BFL) of tolerant (ICCV 92944, ICC 1356, ICC 15614) and sensitive (ICC 10685, ICC 4567, ICC 5912) chickpea genotypes.
**Table S11** Log_10_‐transformed FPKM values of control and heat‐stressed before flowering root tissues (BFR) of tolerant (ICCV 92944, ICC 1356, ICC 15614) and sensitive (ICC 10685, ICC 4567, ICC 5912) chickpea genotypes.
**Table S12** Log_10_‐transformed FPKM values of reproductive stage control and stressed root tissues (AFR) of heat‐sensitive (ICC 10685 and ICC 5912) chickpea genotypes.
**Table S13** Log_10_‐transformed FPKM values of control and heat‐stressed after flowering leaf tissues (AFL) of tolerant (ICCV 92944, ICC 1356, ICC 15614) and sensitive (ICC 10685, ICC 4567, ICC 5912) chickpea genotypes.
**Table S14** Log_10_‐transformed FPKM values of control (nonstressed) vegetative (BFL, BFR) and reproductive stage (AFL, AFF) tissues of tolerant (ICCV 92944, ICC 1356, ICC 15614) and sensitive (ICC 10685, ICC 4567, ICC 5912) chickpea genotypes.
**Table S15** List of primers used for qRT‐PCR in chickpea.
**Table S16** Log_10_‐transformed FPKM values of Fusarium wilt‐stressed resistant pigeonpea genotypes (ICPL 87119, ICPL 99050, ICPW 94) and six sterility mosaic disease‐infected genotypes (six resistant, ICPL 20096, ICPL 7035, BSMR 736) and four sterility mosaic disease‐susceptible genotypes (TTB 7, TAT 10, ICPL 332, ICPB 2049).
**Table S17** List of primers used for qRT‐PCR in pigeonpea.
**Table S18** Summary of chickpea AP2/ERF sequence annotation using Blast2GO.
**Table S19** Summary of pigeonpea AP2/ERF sequence annotation using Blast2GO.
**Table S20** Summary of Medicago AP2/ERF sequence annotation using Blast2GO.
**Table S21** Summary of common bean AP2/ERF sequence annotation using Blast2GO.
**Table S22** Summary of Lotus AP2/ERF sequence annotation using Blast2GO.

**Appendix S1** Experimental procedures, results and discussion.
