# Supplementary material for: Genome‐wide dissection of AP2/ERF and HSP90 gene families in five legumes and expression profiles in chickpea and pigeonpea
Source: Plant Biotechnol J. 2016 Jan 23;14(7):1563–77. doi: 10.1111/pbi.12520 (PMC5066796; doi:10.1111/pbi.12520)
Supplement: Supplementary file 1 — Figure S1 Genome‐wide distribution of AP2/ERF and HSP90 genes in chickpea. A total of 128 AP2/ERF and three HSP90 genes were found to be anchored onto the pseudomolecules, while the remaining (19 AP2/ERF and two HSP90) genes were localized on the scaffolds. Clusters of tandemly duplicated genes are highlighted in green and those linked with lines represent segmentally duplicated genes. [file PBI-14-1563-s001.pdf]

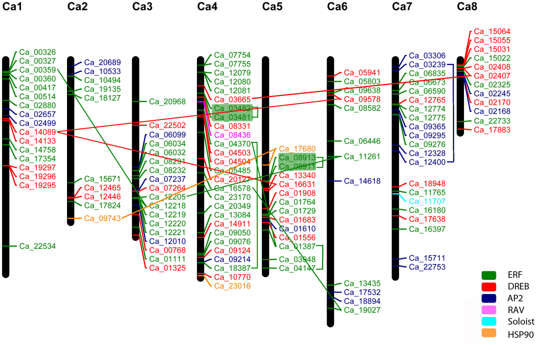

Supplementary Figure 1. Genome-wide distribution of AP2/ERF and HSP90 genes in chickpea.
A total of 128 AP2/ERF and 3 HSP90 genes were found to be anchored onto the pseudomolecules, while the remaining (19 AP2/ERF and 2 HSP) genes were localized on the scaffolds. Clusters of tandemly duplicated genes are highlighted in green and those linked with lines represent segmentally duplicated genes.
