# Supplementary figures and images for: Genome‐wide dissection of AP2/ERF and HSP90 gene families in five legumes and expression profiles in chickpea and pigeonpea
Source: Plant Biotechnol J. 2016 Jan 23;14(7):1563–77. doi: 10.1111/pbi.12520 (PMC5066796; doi:10.1111/pbi.12520)

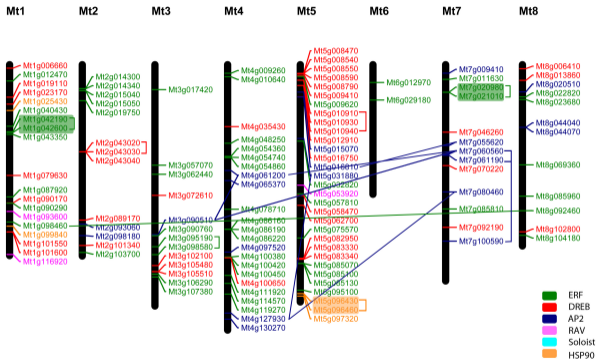

Supplementary Figure 3. Genome wide distribution of AP2/ERF and HSP90 genes in Medicago

Supplement: Supplementary file 3 — Figure S3 Genome‐wide distribution of AP2/ERF and HSP90 genes in Medicago. [file PBI-14-1563-s015.pdf]

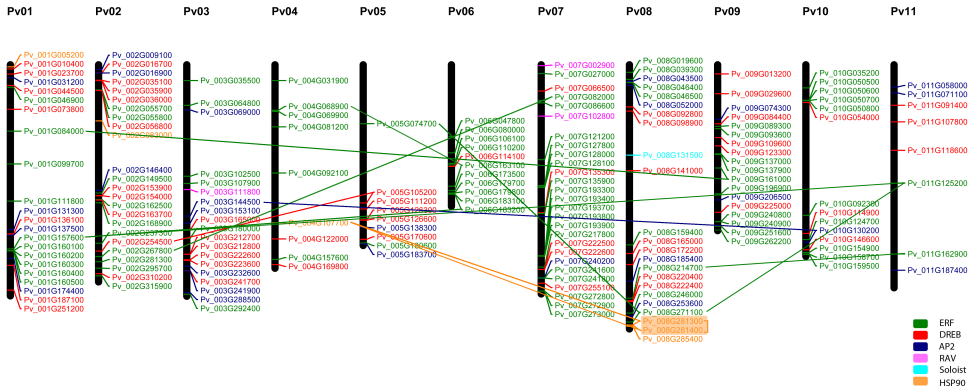

Supplementary Figure 4. Genome wide distribution of AP2/ERF and HSP90 genes in common bean

Supplement: Supplementary file 4 — Figure S4 Genome‐wide distribution of AP2/ERF and HSP90 genes in common bean. [file PBI-14-1563-s017.pdf]

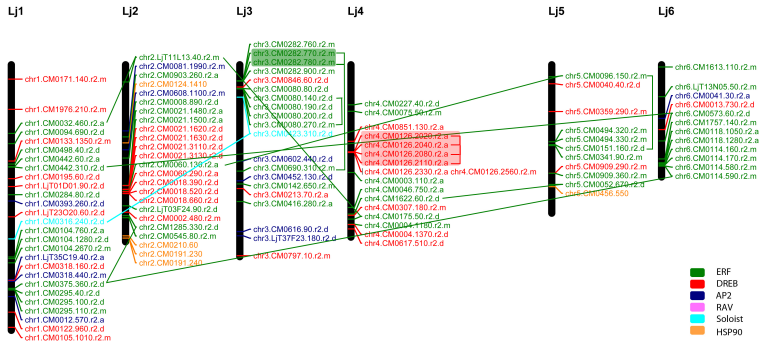

Supplementary Figure 5. Genome wide distribution of AP2/ERF and HSP90 genes in Lotus

Supplement: Supplementary file 5 — Figure S5 Genome‐wide distribution of AP2/ERF and HSP90 genes in Lotus. [file PBI-14-1563-s018.pdf]

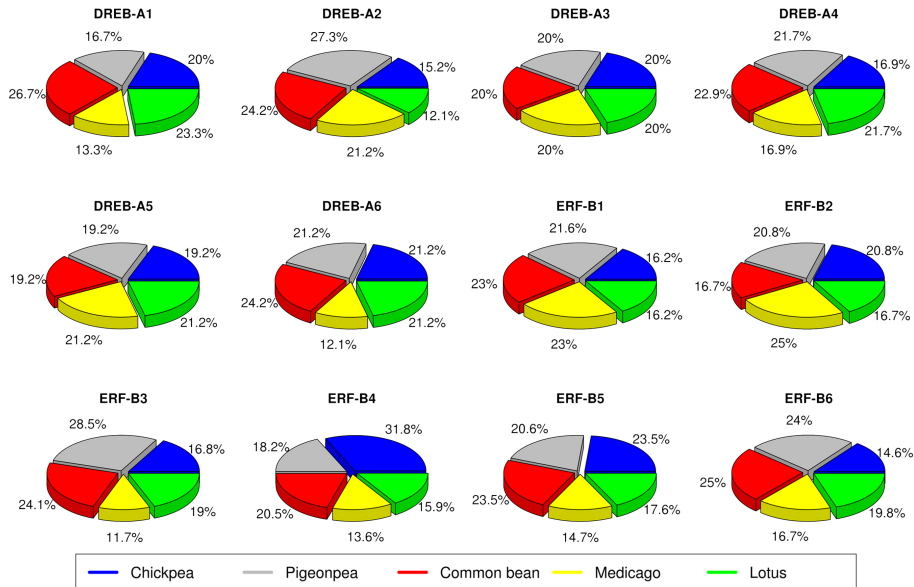

Supplementary Figure 6. Pie-chart representation of DREB (A1 to A6) and ERF (B1 to B6) genes in five legumes

Supplement: Supplementary file 6 — Figure S6 Pie chart representation of DREB (A1–A6) and ERF (B1–B6) genes in five legumes. [file PBI-14-1563-s002.pdf]

**a**

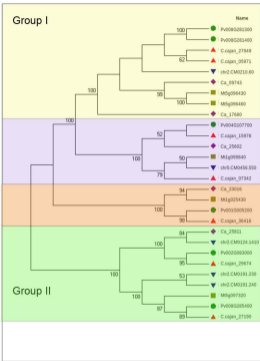

**b**

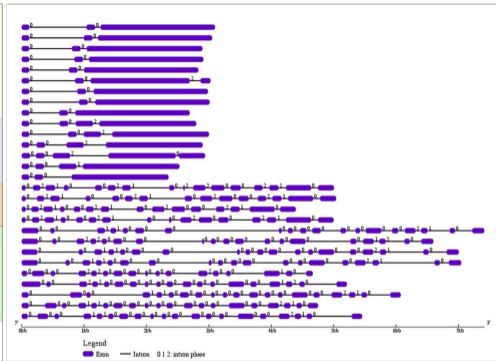

**C**

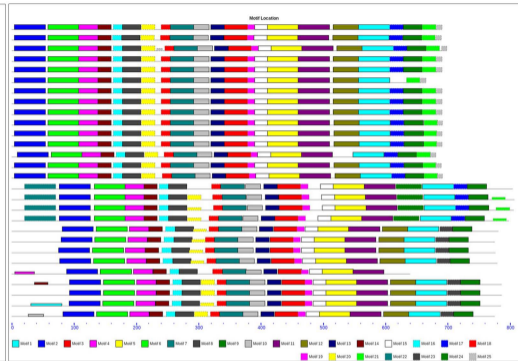

Supplement: Supplementary file 10 — Figure S10 Phylogenetic relationships, gene structures and motif composition of HSP90 genes in chickpea (Ca), pigeonpea (Cc), common bean (Pv), Medicago (Mt) and Lotus (Lj). (a) Phylogenetic tree constructed using MEGA 5.0 by neighbour‐joining (NJ) method with 1000 bootstrap replicates. Bootstrap support is indicated at each node. (b) Exon/intron structures of the HSP90 genes. Blue boxes represent exons and black lines represent introns. The numbers indicate the splicing phases of HSP90 genes: 0, phase 0; 1, phase 1; and 2, phase 2. (c) Schematic representation of conserved motifs (obtained using MEME) in HSP90 proteins. Different motifs are represented by boxes of different colours. [file PBI-14-1563-s006.pdf]

Motif Overview

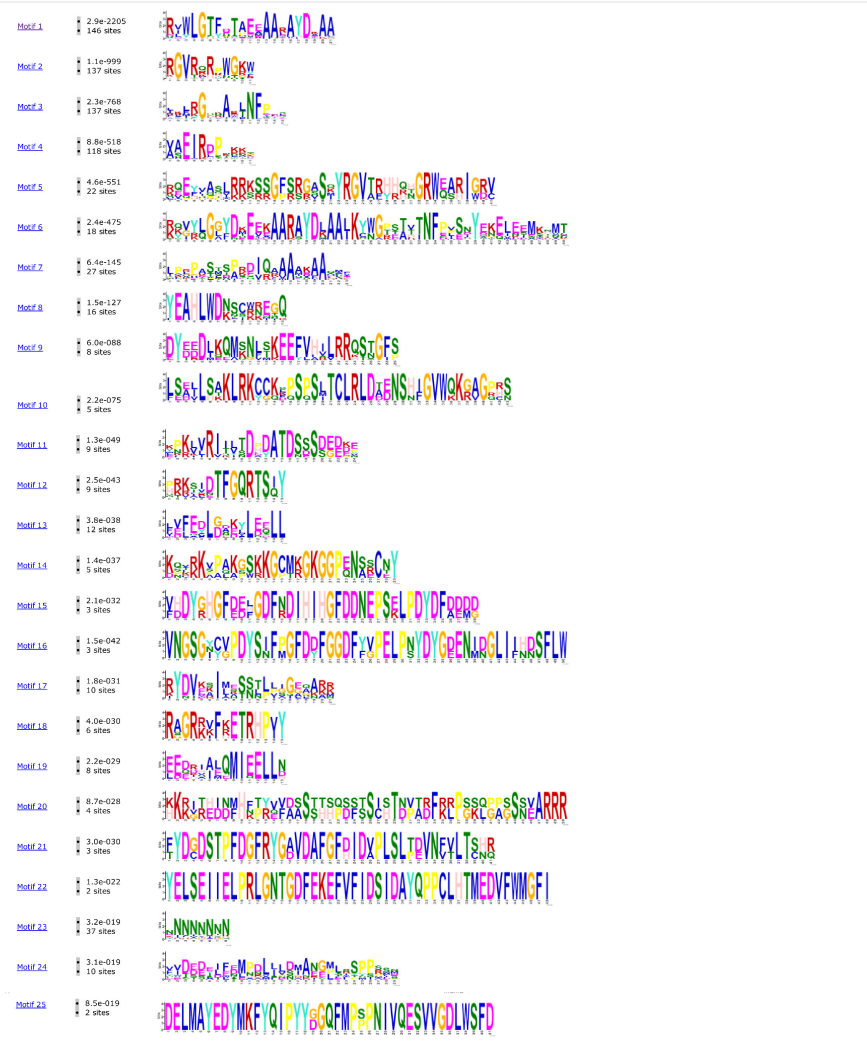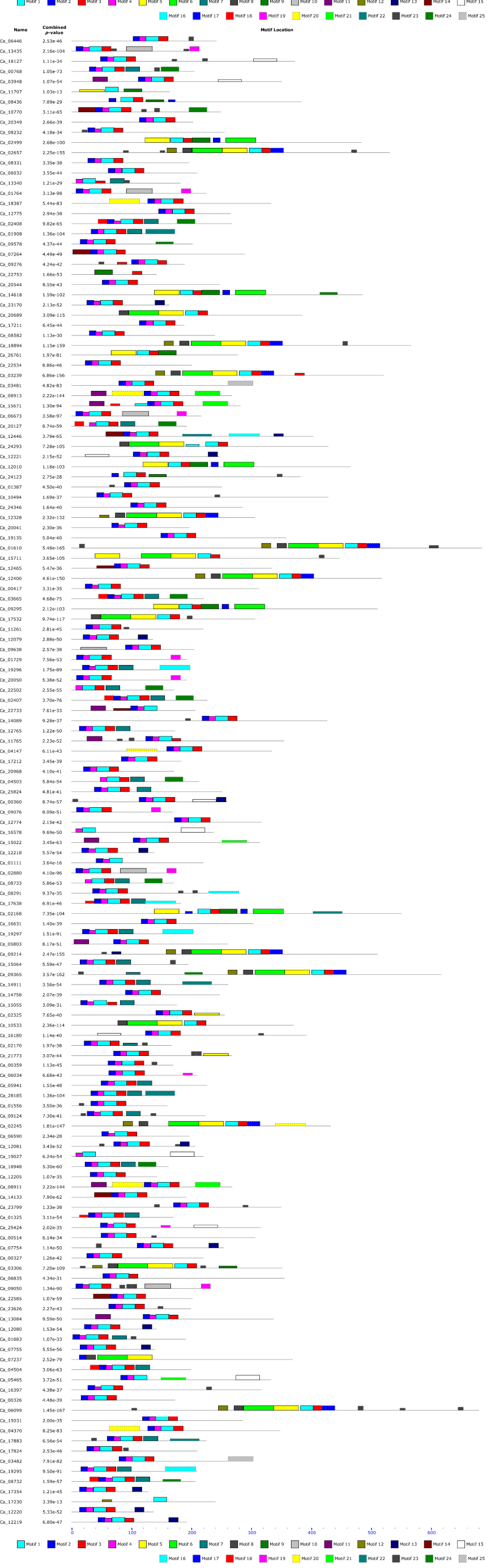

Supplementary Figure 11. Putative motif prediction in chickpea using MEME

Supplement: Supplementary file 11 — Figure S11 Putative motif prediction in chickpea using MEME. [file PBI-14-1563-s007.pdf]

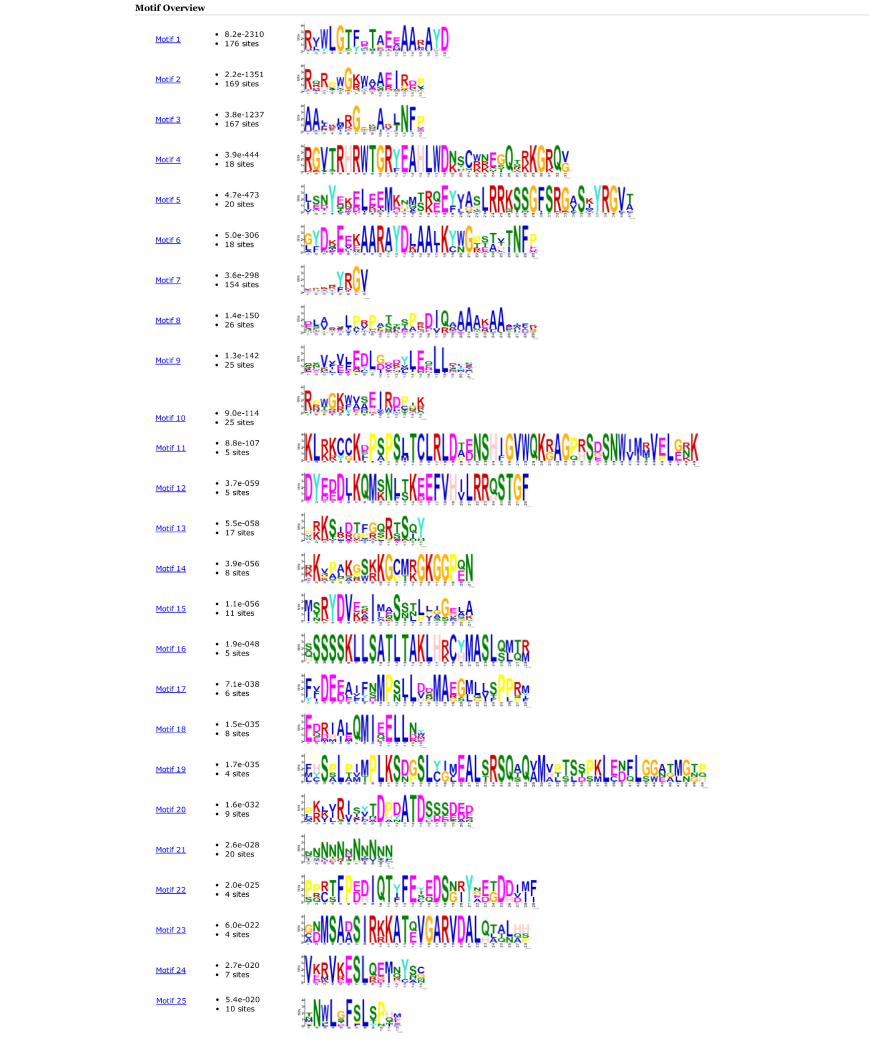

Supplement: Supplementary file 12 — Figure S12 Putative motif prediction in pigeonpea using MEME. [file PBI-14-1563-s008.pdf]

Motif Overview

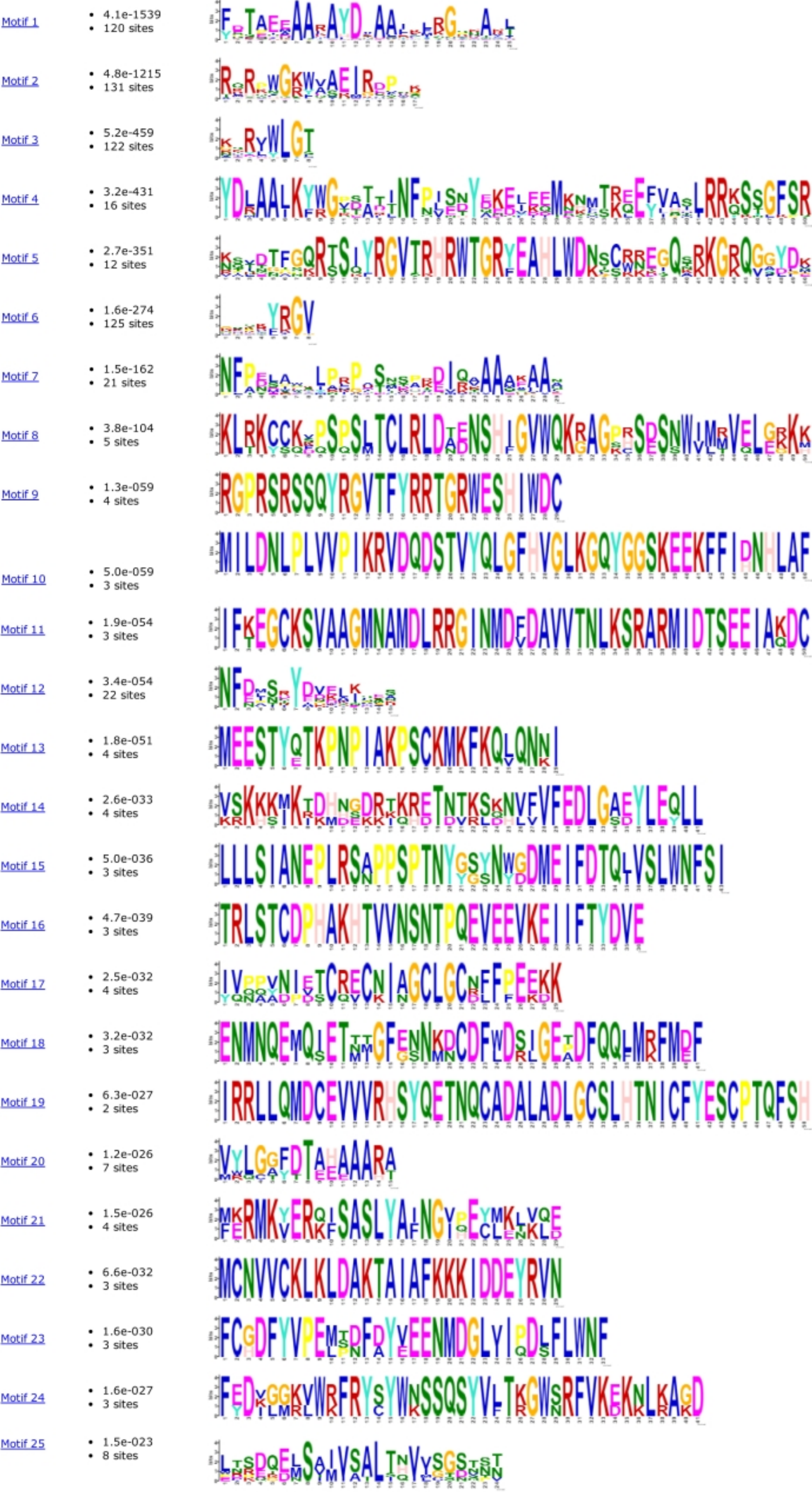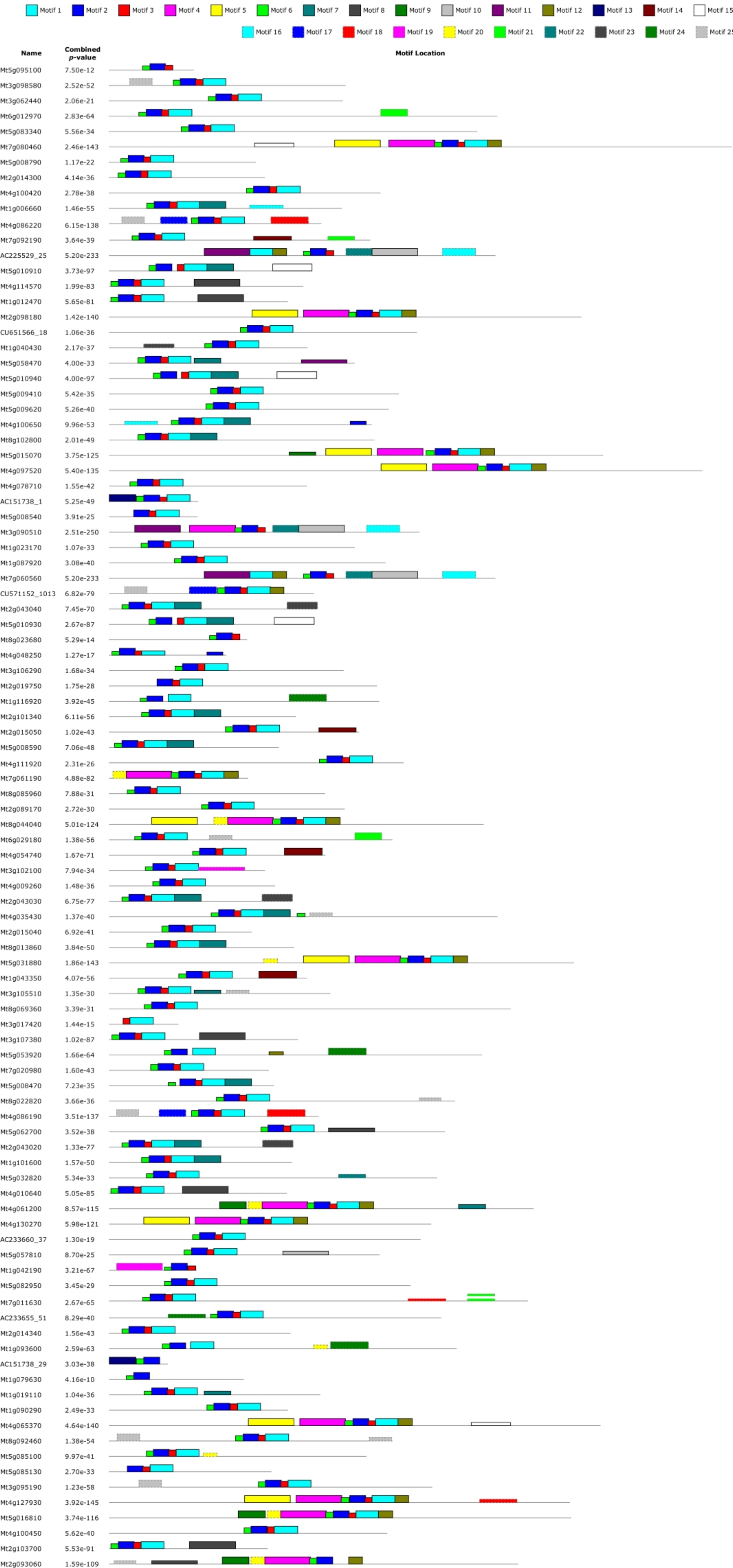

Supplement: Supplementary file 13 — Figure S13 Putative motif prediction in Medicago using MEME. [file PBI-14-1563-s009.pdf]
