# Supplementary material for: Genome‐wide dissection of AP2/ERF and HSP90 gene families in five legumes and expression profiles in chickpea and pigeonpea
Source: Plant Biotechnol J. 2016 Jan 23;14(7):1563–77. doi: 10.1111/pbi.12520 (PMC5066796; doi:10.1111/pbi.12520)
Supplement: Supplementary file 8 — Figure S8 Phylogenetic tree based on conserved domain sequence of AP2/ERF protein in common bean. The unrooted tree was divided into 12 groups, ERF (marked in green), DREB (marked in red), AP2 (marked in blue), RAV (marked in pink) and soloist (marked in teak). Legends on the right represent the respective subfamily members. Only bootstrap values greater than 50% support are indicated. [file PBI-14-1563-s004.pdf]

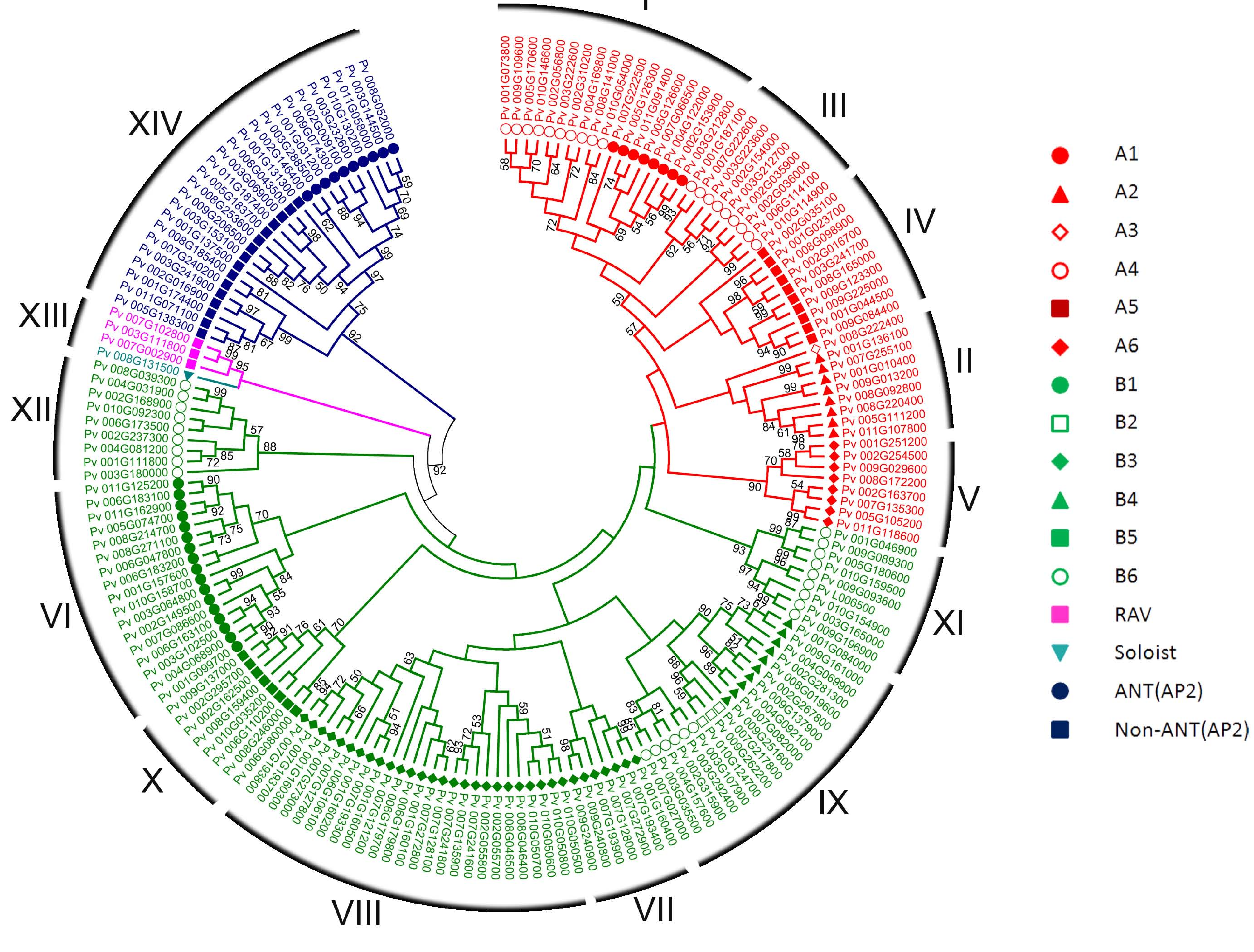

Supplementary Figure 8. Phylogenetic tree based on conserved domain sequence of AP2/ERF protein in common bean. The unrooted tree was divided into twelve groups, ERF (marked in green), DREB (marked in red), AP2 (marked in blue), RAV (marked in pink) and soloist (marked in teak). Legends on the right represent the respective subfamily members. Only bootstrap values greater than 50% support are indicated.
