# Supplementary material for: Genome‐wide dissection of AP2/ERF and HSP90 gene families in five legumes and expression profiles in chickpea and pigeonpea
Source: Plant Biotechnol J. 2016 Jan 23;14(7):1563–77. doi: 10.1111/pbi.12520 (PMC5066796; doi:10.1111/pbi.12520)
Supplement: Supplementary file 14 — Figure S14 Putative motif prediction in common bean using MEME. [file PBI-14-1563-s010.pdf]

Motif Overview

Motif	E-value / sites	Logo
Motif 1	• 1.4e-2717 • 179 sites	
Motif 2	• 4.4e-1459 • 177 sites	
Motif 3	• 1.3e-990 • 171 sites	
Motif 4	• 4.0e-507 • 17 sites	
Motif 5	• 1.5e-400 • 24 sites	
Motif 6	• 2.3e-398 • 18 sites	
Motif 7	• 1.4e-321 • 155 sites	
Motif 8	• 1.9e-178 • 29 sites	
Motif 9	• 5.5e-123 • 20 sites	
Motif 10	• 2.1e-077 • 9 sites	
Motif 11	• 6.8e-075 • 21 sites	
Motif 12	• 1.1e-073 • 4 sites	
Motif 13	• 3.9e-059 • 8 sites	
Motif 14	• 3.6e-056 • 12 sites	
Motif 15	• 1.9e-044 • 9 sites	
Motif 16	• 5.0e-036 • 5 sites	
Motif 17	• 2.2e-031 • 3 sites	
Motif 18	• 2.1e-031 • 5 sites	
Motif 19	• 7.9e-029 • 10 sites	
Motif 20	• 5.7e-028 • 4 sites	
Motif 21	• 5.1e-024 • 4 sites	
Motif 22	• 7.4e-026 • 4 sites	
Motif 23	• 6.7e-022 • 5 sites	
Motif 24	• 2.3e-021 • 4 sites	
Motif 25	• 2.5e-021 • 6 sites	

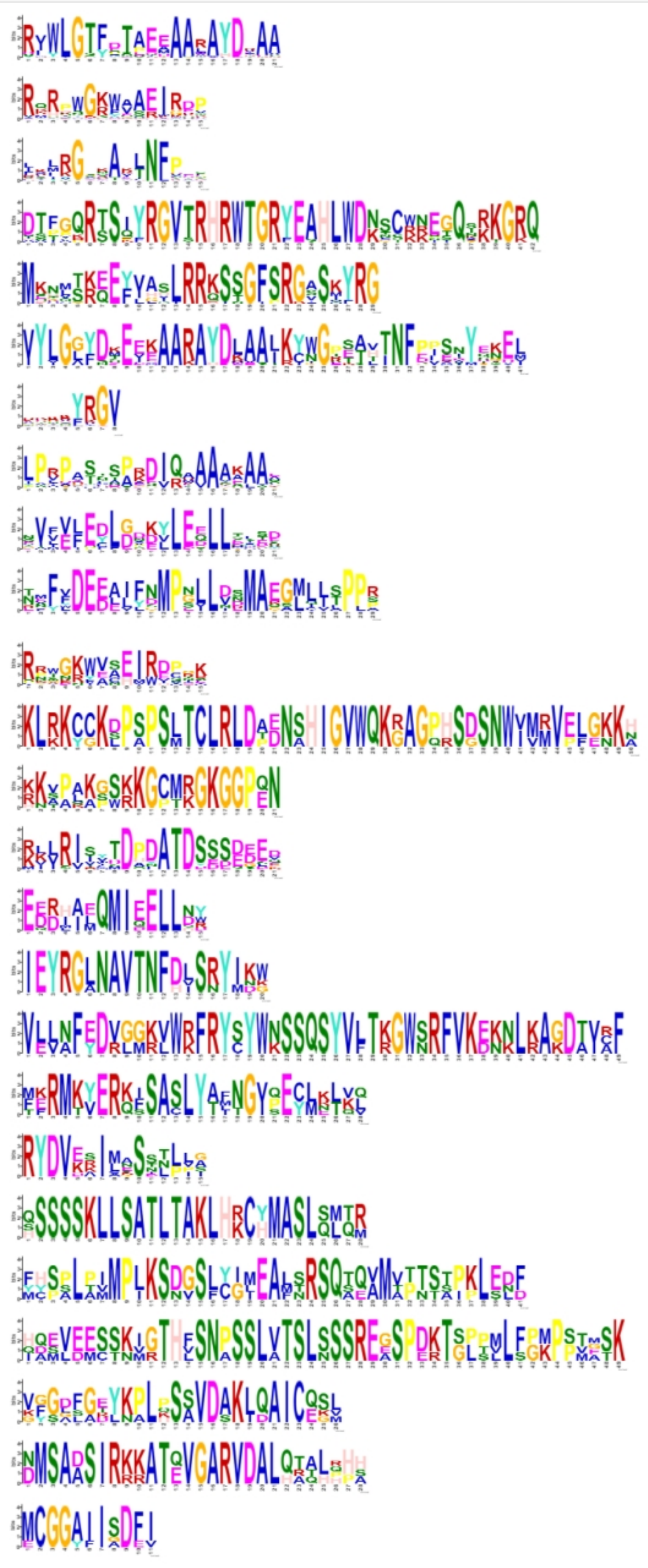

Combined Block Diagrams

Non-overlapping sites with a p-value better than 0.0001.
The height of the motif "block" is proportional to -log(p-value), truncated at the height for a motif with a p-value of 1e-10.
Click on any row to highlight sequence in all motifs. The motif blocks have tool tips with more information.

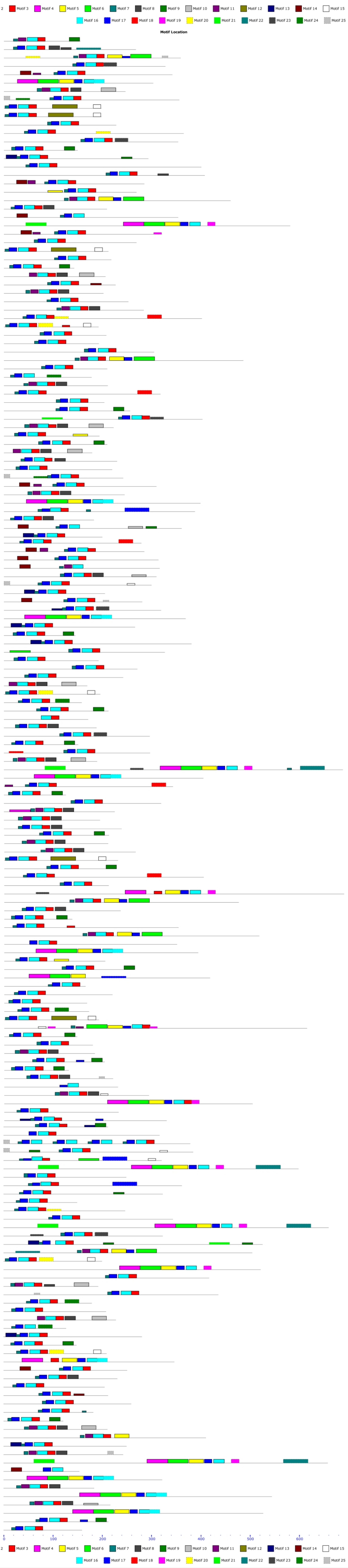

Name	Combined p-value
Pv_007G121200	9.80e-58
Pv_009G084400	5.70e-51
Pv_008G185400	1.46e-64
Pv_008G172200	4.74e-62
Pv_009G137000	1.32e-64
Pv_008G253600	5.29e-134
Pv_005G126300	7.72e-74
Pv_009G262200	2.99e-64
Pv_009G093600	3.55e-114
Pv_010G154900	1.28e-116
Pv_003G292400	8.88e-59
Pv_003G064800	4.66e-42
Pv_009G029600	7.23e-70
Pv_007G272800	3.96e-59
Pv_007G255100	7.68e-60
Pv_002G149500	9.30e-40
Pv_005G105200	2.81e-45
Pv_006G110200	1.27e-61
Pv_007G241600	3.48e-56
Pv_001G174400	5.52e-91
Pv_009G225000	7.47e-43
Pv_007G027000	9.71e-37
Pv_010G130200	4.94e-157
Pv_008G159400	7.56e-64
Pv_007G082000	3.03e-54
Pv_009G196900	2.15e-107
Pv_010G050500	5.33e-43
Pv_007G128000	3.96e-62
Pv_003G223600	7.39e-67
Pv_002G281300	1.71e-54
Pv_007G066500	8.04e-57
Pv_003G180000	3.68e-39
Pv_010G054000	3.88e-65
Pv_008G039300	2.30e-68
Pv_009G089300	9.97e-90
Pv_008G046400	9.83e-52
Pv_010G050600	4.41e-49
Pv_002G055800	7.41e-53
Pv_011G071100	5.73e-95
Pv_010G158700	3.41e-43
Pv_002G035100	2.56e-34
Pv_005G170600	3.21e-59
Pv_004G081200	1.40e-59
Pv_007G135900	1.26e-53
Pv_007G193300	5.54e-57
Pv_007G135300	6.15e-47
Pv_011G091400	2.09e-68
Pv_005G074700	1.04e-56
Pv_007G127800	8.29e-62
Pv_003G212700	3.32e-71
Pv_001G044500	8.99e-46
Pv_008G271100	5.37e-48
Pv_009G251600	3.01e-62
Pv_002G295700	5.49e-64
Pv_001G073800	8.20e-64
Pv_011G187400	3.69e-122
Pv_003G111800	3.29e-83
Pv_002G035900	1.13e-55
Pv_003G035500	2.01e-44
Pv_011G107800	1.18e-57
Pv_001G111800	1.44e-62
Pv_010G035200	4.96e-59
Pv_002G162500	5.15e-63
Pv_004G157600	3.54e-41
Pv_008G141000	4.35e-63
Pv_007G217800	6.11e-63
Pv_005G111200	9.75e-61
Pv_008G246000	6.65e-69
Pv_002G254500	1.47e-68
Pv_003G153100	3.06e-133
Pv_001G010400	7.89e-57
Pv_007G272900	5.41e-65
Pv_008G220400	1.05e-60
Pv_001G084000	2.75e-53
Pv_011G162900	6.27e-52
Pv_002G055700	3.21e-52
Pv_008G019600	3.11e-55
Pv_002G154000	5.57e-68
Pv_010G159500	1.05e-86
Pv_008G098900	7.52e-72
Pv_007G193700	2.62e-64
Pv_001G160300	1.34e-26
Pv_002G056800	1.25e-65
Pv_001G251200	3.01e-71
Pv_001G160400	1.06e-60
Pv_009G137900	2.29e-55
Pv_001G187100	1.02e-84
Pv_001G031200	8.90e-241
Pv_003G069000	3.82e-128
Pv_002G168900	2.08e-59
Pv_009G240800	1.10e-64
Pv_002G267800	4.19e-49
Pv_004G122000	4.00e-55
Pv_009G109600	1.30e-65
Pv_008G165000	5.98e-47
Pv_007G193800	4.56e-65
Pv_010G146600	5.10e-61
Pv_002G036000	3.52e-52
Pv_003G165000	1.13e-102
Pv_001G160500	3.72e-58
Pv_004G031900	4.21e-66
Pv_007G241800	1.06e-55
Pv_008G052000	3.46e-141
Pv_005G138300	3.52e-94
Pv_009G123300	8.03e-48
Pv_007G193900	1.23e-70
Pv_006G173500	2.58e-40
Pv_003G241900	1.78e-93
Pv_003G102500	5.85e-43
Pv_008G043500	6.00e-130
Pv_008G214700	6.98e-50
Pv_006G179700	6.86e-63
Pv_001G131300	2.58e-98
Pv_010G050800	4.80e-47
Pv_006G183100	3.18e-53
Pv_006G183200	2.81e-46
Pv_002G016700	4.89e-76
Pv_L006500	2.66e-110
Pv_001G137500	5.37e-43
Pv_007G193400	5.57e-59
Pv_006G179800	4.64e-50
Pv_006G114100	7.42e-62
Pv_006G106100	1.81e-63
Pv_001G160100	1.05e-68
Pv_004G169800	1.30e-65
Pv_008G131500	5.57e-10
Pv_003G212800	7.20e-55
Pv_002G146400	1.47e-149
Pv_001G099700	1.01e-39
Pv_008G222400	3.10e-43
Pv_001G160200	6.10e-58
Pv_006G163100	5.52e-39
Pv_003G107900	2.75e-53
Pv_010G124700	1.44e-61
Pv_007G002900	2.68e-54
Pv_002G009100	1.32e-217
Pv_004G069900	5.79e-55
Pv_007G102800	5.51e-78
Pv_010G092300	3.58e-40
Pv_007G086600	8.45e-41
Pv_002G237300	1.41e-35
Pv_004G068900	1.57e-40
Pv_003G232600	5.95e-233
Pv_002G163700	9.36e-59
Pv_008G092800	1.10e-62
Pv_002G016900	2.58e-96
Pv_001G046900	2.96e-86
Pv_003G288500	2.57e-136
Pv_011G118600	1.05e-44
Pv_007G222500	7.18e-51
Pv_004G092100	1.94e-54
Pv_001G023700	1.74e-74
Pv_006G047800	2.24e-48
Pv_005G126600	2.38e-69
Pv_003G241700	7.30e-52
Pv_001G136100	2.13e-61
Pv_007G128100	1.09e-63
Pv_005G180600	2.06e-83
Pv_009G206500	6.09e-130
Pv_006G080000	3.89e-58
Pv_003G222600	2.34e-60
Pv_011G125200	9.52e-52
Pv_010G050700	2.05e-47
Pv_009G161000	7.73e-55
Pv_008G046500	1.35e-41
Pv_009G240900	1.39e-65
Pv_007G222600	2.87e-83
Pv_007G240200	3.65e-71
Pv_009G013200	5.20e-58
Pv_002G310200	4.28e-70
Pv_009G074300	4.20e-227
Pv_002G315900	2.81e-39
Pv_005G183700	4.67e-127
Pv_010G114900	1.05e-58
Pv_011G058000	7.79e-152
Pv_002G153900	9.48e-52
Pv_003G144500	1.16e-154
Pv_007G273000	3.91e-65
Pv_001G157600	1.09e-43
