# Supplementary material for: Genome‐wide dissection of AP2/ERF and HSP90 gene families in five legumes and expression profiles in chickpea and pigeonpea
Source: Plant Biotechnol J. 2016 Jan 23;14(7):1563–77. doi: 10.1111/pbi.12520 (PMC5066796; doi:10.1111/pbi.12520)
Supplement: Supplementary file 15 — Figure S15 Putative motif prediction in Lotus using MEME. [file PBI-14-1563-s011.pdf]

Motif Overview

Motif	E-value	Sites
Motif 1	6.8e-2432	135 sites
Motif 2	8.1e-1285	119 sites
Motif 3	3.5e-233	118 sites
Motif 4	1.0e-223	13 sites
Motif 5	1.6e-197	117 sites
Motif 6	8.4e-138	6 sites
Motif 7	2.5e-126	25 sites
Motif 8	6.1e-126	10 sites
Motif 9	6.1e-070	4 sites
Motif 10	1.4e-053	4 sites
Motif 11	2.1e-051	6 sites
Motif 12	5.8e-046	5 sites
Motif 13	4.3e-043	9 sites
Motif 14	3.7e-031	11 sites
Motif 15	6.9e-030	4 sites
Motif 16	1.5e-031	3 sites
Motif 17	1.3e-039	3 sites
Motif 18	8.8e-037	13 sites
Motif 19	3.5e-023	2 sites
Motif 20	5.1e-023	5 sites
Motif 21	2.4e-020	21 sites
Motif 22	4.1e-018	3 sites
Motif 23	2.7e-017	4 sites
Motif 24	1.9e-017	3 sites

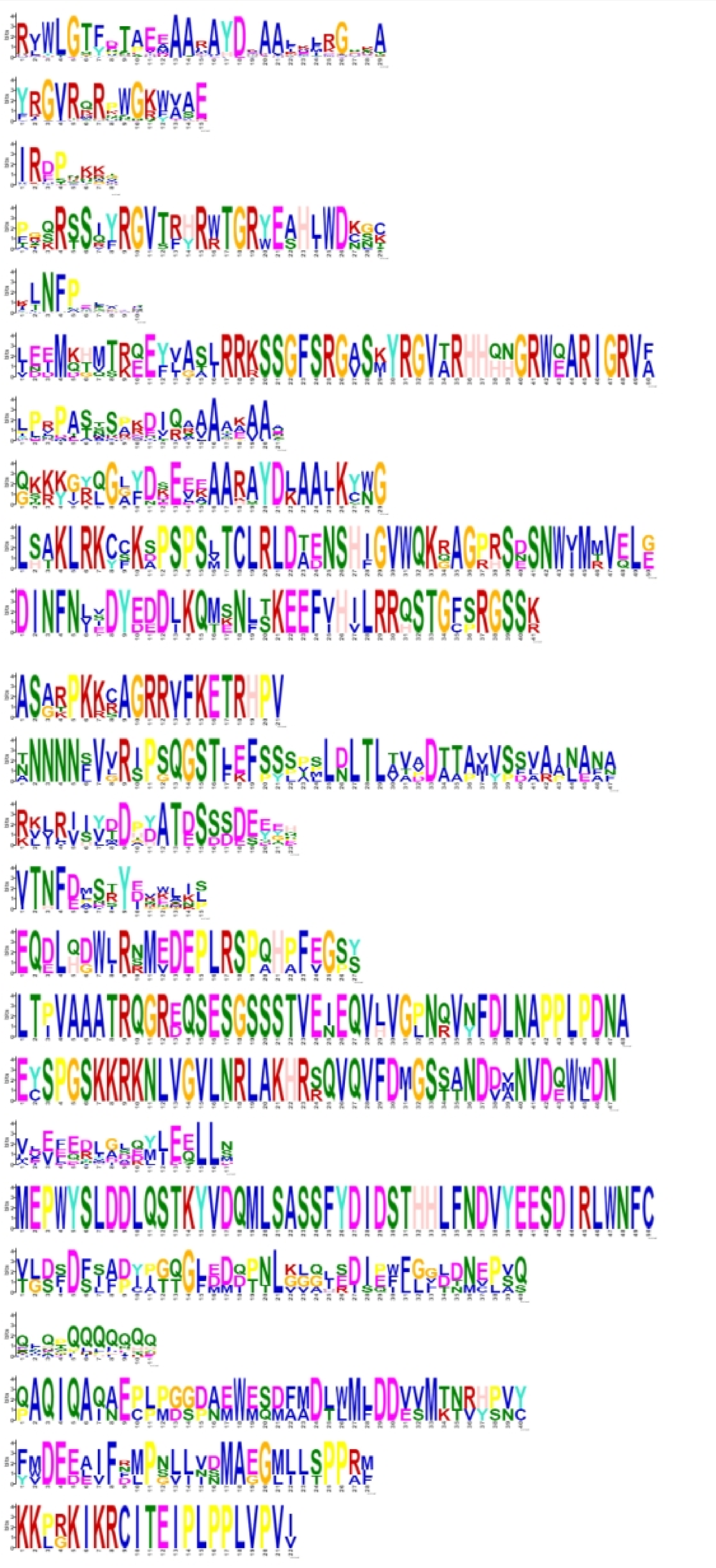

Combined Block Diagrams

Non-overlapping sites with a *p*-value better than 0.0001.
The height of the motif "block" is proportional to -log(p-value), truncated at the height for a motif with a p-value of 1e-10.
Click on any row to highlight sequence in all motifs. The motif blocks have tool tips with more information.

Motif 1, Motif 2, Motif 3, Motif 4, Motif 5, Motif 6, Motif 7, Motif 8, Motif 9, Motif 10, Motif 11, Motif 12, Motif 13, Motif 14, Motif 15, Motif 16, Motif 17, Motif 18, Motif 19, Motif 20, Motif 21, Motif 22, Motif 23, Motif 24, Motif 25

Name	Combined *p*-value
LjSGA_027251.1	1.30e-46
LjSGA_016572.1	7.24e-101
chr3.CM0416.280.r2.a	2.01e-39
chr6.CM0013.730.r2.d	3.46e-34
chr4.CM0126.2080.r2.a	6.10e-93
chr2.CM0021.3110.r2.d	1.19e-73
chr1.CM0104.1280.r2.d	3.76e-35
chr5.CM0494.320.r2.m	2.45e-58
LjSGA_051932.1	3.00e-50
LjSGA_005350.1	5.78e-40
chr1.CM0105.1010.r2.m	2.35e-49
chr1.CM0393.260.r2.d	9.26e-145
LjSGA_063888.1	5.05e-41
chr4.CM0175.50.r2.d	4.83e-40
chr2.LjT11L13.40.r2.m	3.42e-48
chr2.CM0060.130.r2.a	6.52e-50
LjSGA_107677.3	4.75e-35
LjSGA_005424.1	1.55e-126
chr3.CM0142.650.r2.m	3.90e-102
LjSGA_146586.1	9.47e-40
chr2.CM0018.520.r2.d	2.13e-50
chr2.CM0018.660.r2.d	4.47e-64
chr6.CM0114.160.r2.m	5.27e-56
LjSGA_147935.1	4.58e-26
chr1.CM0295.40.r2.d	7.01e-52
chr3.CM0690.310.r2.m	6.29e-131
chr2.CM0008.890.r2.d	6.69e-31
chr3.CM0846.60.r2.d	1.11e-58
chr2.CM0021.1630.r2.d	1.12e-49
LjSGA_034198.1	5.12e-38
chr1.CM0171.140.r2.m	3.43e-48
chr2.CM0002.480.r2.m	4.79e-36
LjT33A04.100.r2.m	7.85e-49
chr3.CM0797.10.r2.m	6.96e-50
chr6.CM0114.590.r2.m	2.47e-36
LjSGA_017232.1	5.29e-65
chr5.CM0909.360.r2.m	5.79e-39
chr6.CM0573.60.r2.d	3.66e-116
LjSGA_134387.1	1.55e-35
chr1.CM1976.210.r2.m	1.89e-95
chr4.CM0617.510.r2.d	3.61e-51
chr1.CM0284.80.r2.d	1.29e-35
chr5.CM0151.160.r2.d	6.05e-22
chr1.CM0012.570.r2.a	4.97e-121
chr6.CM1613.110.r2.m	4.32e-72
chr2.CM0021.1480.r2.a	1.31e-40
chr4.CM0126.2020.r2.a	3.95e-24
chr5.CM0359.290.r2.m	3.32e-49
chr4.CM0126.2040.r2.a	7.62e-85
chr3.CM0080.200.r2.d	8.74e-87
LjSGA_012098.1	3.10e-82
chr2.CM0021.1620.r2.d	3.56e-53
chr1.CM0442.60.r2.a	1.43e-91
chr1.CM0316.240.r2.d	8.88e-12
LjSGA_026378.1	3.40e-30
chr2.CM0545.80.r2.m	1.99e-80
chr3.CM0282.900.r2.m	1.34e-68
LjT33G24.10.r2.d	3.91e-44
chr3.CM0423.310.r2.d	6.90e-13
chr4.CM0004.1370.r2.d	9.51e-37
LjSGA_028626.1	1.70e-46
chr4.CM1622.60.r2.d	2.54e-42
chr3.LjT37F23.180.r2.d	1.30e-139
chr6.CM0114.580.r2.m	1.67e-43
chr5.CM0040.40.r2.d	1.92e-37
LjSGA_047986.1	3.37e-31
chr1.CM0122.960.r2.d	5.84e-25
chr4.CM0003.110.r2.a	2.87e-100
chr5.CM0341.90.r2.m	2.22e-68
LjSGA_019604.2	4.80e-06
chr3.CM0080.80.r2.d	1.87e-91
chr2.CM0018.390.r2.d	5.46e-26
chr1.CM0104.2670.r2.m	3.33e-37
chr4.CM0075.50.r2.m	9.73e-34
chr1.LjT23O20.60.r2.d	6.80e-45
chr1.LjT35C19.40.r2.a	1.57e-86
LjSGA_025266.1	2.02e-41
chr1.CM0318.440.r2.m	4.88e-37
chr4.CM0126.2110.r2.a	2.96e-97
LjSGA_080421.2	6.84e-25
chr5.CM0909.290.r2.m	1.15e-34
chr3.CM0452.130.r2.d	3.80e-59
chr2.CM1285.330.r2.d	3.14e-36
chr5.CM0096.150.r2.m	1.99e-19
chr5.CM0052.670.r2.d	1.87e-44
chr1.CM0104.760.r2.a	1.36e-43
chr2.CM0608.1100.r2.m	2.65e-131
chr1.CM0032.460.r2.a	2.93e-42
CM0905.90.r2.d	5.16e-43
LjSGA_035548.1	3.23e-74
chr2.LjT03F24.90.r2.d	2.08e-21
chr2.CM0060.290.r2.a	3.29e-40
LjSGA_007156.1	2.26e-52
chr1.CM0195.60.r2.d	5.73e-35
chr3.CM0080.270.r2.m	2.59e-52
LjT35E05.90.r2.d	1.89e-95
chr1.LjT01D01.90.r2.d	1.43e-31
chr2.CM0903.260.r2.a	3.00e-49
LjSGA_008718.1	1.61e-51
chr4.CM0046.750.r2.a	3.00e-30
chr3.CM0282.780.r2.m	1.14e-125
chr2.CM0081.1990.r2.m	1.54e-141
chr1.CM0295.100.r2.d	1.40e-35
chr3.CM0213.70.r2.a	2.25e-50
chr2.CM0021.1500.r2.a	3.00e-42
chr6.CM0118.1050.r2.a	6.27e-31
chr6.CM0118.1280.r2.a	2.74e-26
chr4.CM0126.2330.r2.a	6.71e-79
chr3.CM0080.140.r2.d	4.07e-74
LjSGA_004289.2	2.40e-33
chr4.CM0307.180.r2.m	2.98e-44
chr2.CM0021.3130.r2.d	1.06e-56
LjSGA_005349.2	6.26e-42
chr4.CM0126.2560.r2.m	2.49e-66
chr3.CM0282.770.r2.m	3.05e-124
LjSGA_007705.1	2.18e-33
chr4.CM0004.1180.r2.m	6.75e-46
chr1.CM0295.110.r2.m	1.95e-62
chr4.CM0227.40.r2.d	1.72e-40
chr3.CM0616.90.r2.d	1.14e-125
chr1.CM0498.40.r2.d	2.29e-43
LjT39B13.80.r2.m	5.42e-128
chr6.CM0041.30.r2.a	1.04e-133
chr3.CM0602.440.r2.d	1.14e-143
chr1.CM0133.1350.r2.m	1.73e-37
LjSGA_003590.1.1	3.36e-07
chr1.CM0318.160.r2.d	1.51e-34
chr6.CM0114.170.r2.m	1.33e-48
chr6.CM1757.140.r2.m	1.07e-31
chr3.CM0080.190.r2.d	3.89e-40
chr5.CM0494.330.r2.m	1.60e-32
chr1.CM0442.310.r2.d	3.88e-117
LjSGA_031594.1	4.16e-17
chr3.CM0282.760.r2.m	9.51e-67
chr1.CM0375.360.r2.d	1.62e-36
chr4.CM0851.130.r2.a	1.27e-51
LjSGA_141699.1	7.68e-42
chr6.LjT13N05.50.r2.m	9.52e-85
chr1.CM0094.690.r2.d	2.07e-44
LjSGA_100683.2.1	4.27e-09

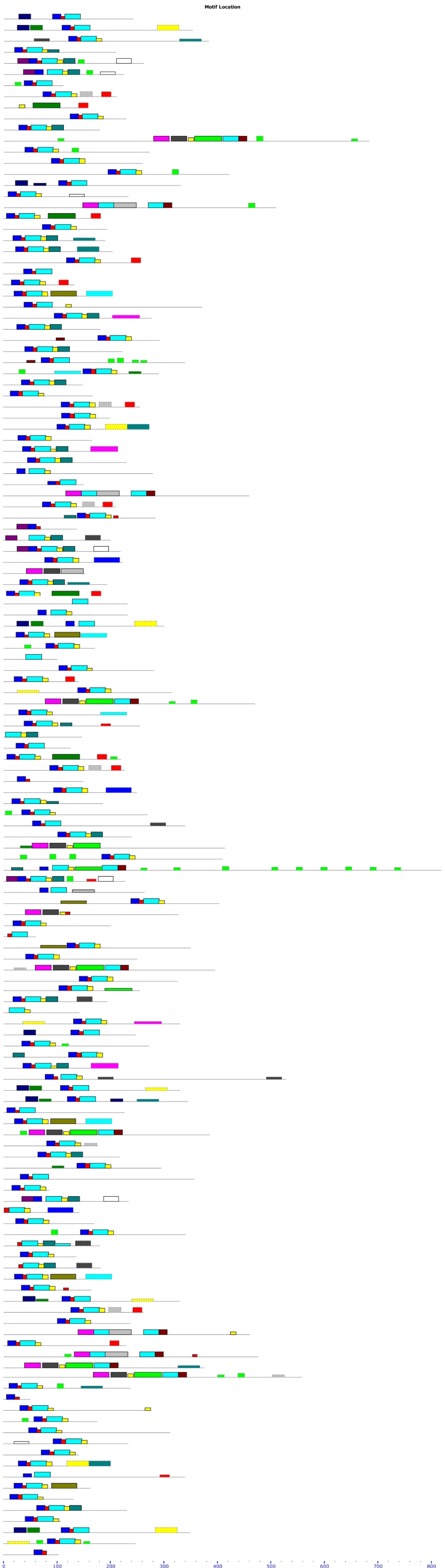

Motif 1, Motif 2, Motif 3, Motif 4, Motif 5, Motif 6, Motif 7, Motif 8, Motif 9, Motif 10, Motif 11, Motif 12, Motif 13, Motif 14, Motif 15, Motif 16, Motif 17, Motif 18, Motif 19, Motif 20, Motif 21, Motif 22, Motif 23, Motif 24, Motif 25
