# Supplementary material for: Genome‐wide dissection of AP2/ERF and HSP90 gene families in five legumes and expression profiles in chickpea and pigeonpea
Source: Plant Biotechnol J. 2016 Jan 23;14(7):1563–77. doi: 10.1111/pbi.12520 (PMC5066796; doi:10.1111/pbi.12520)
Supplement: Supplementary file 16 — Figure S16 Gene ontology assignment to the AP2/ERF sequences identified in the five legumes. [file PBI-14-1563-s012.pdf]

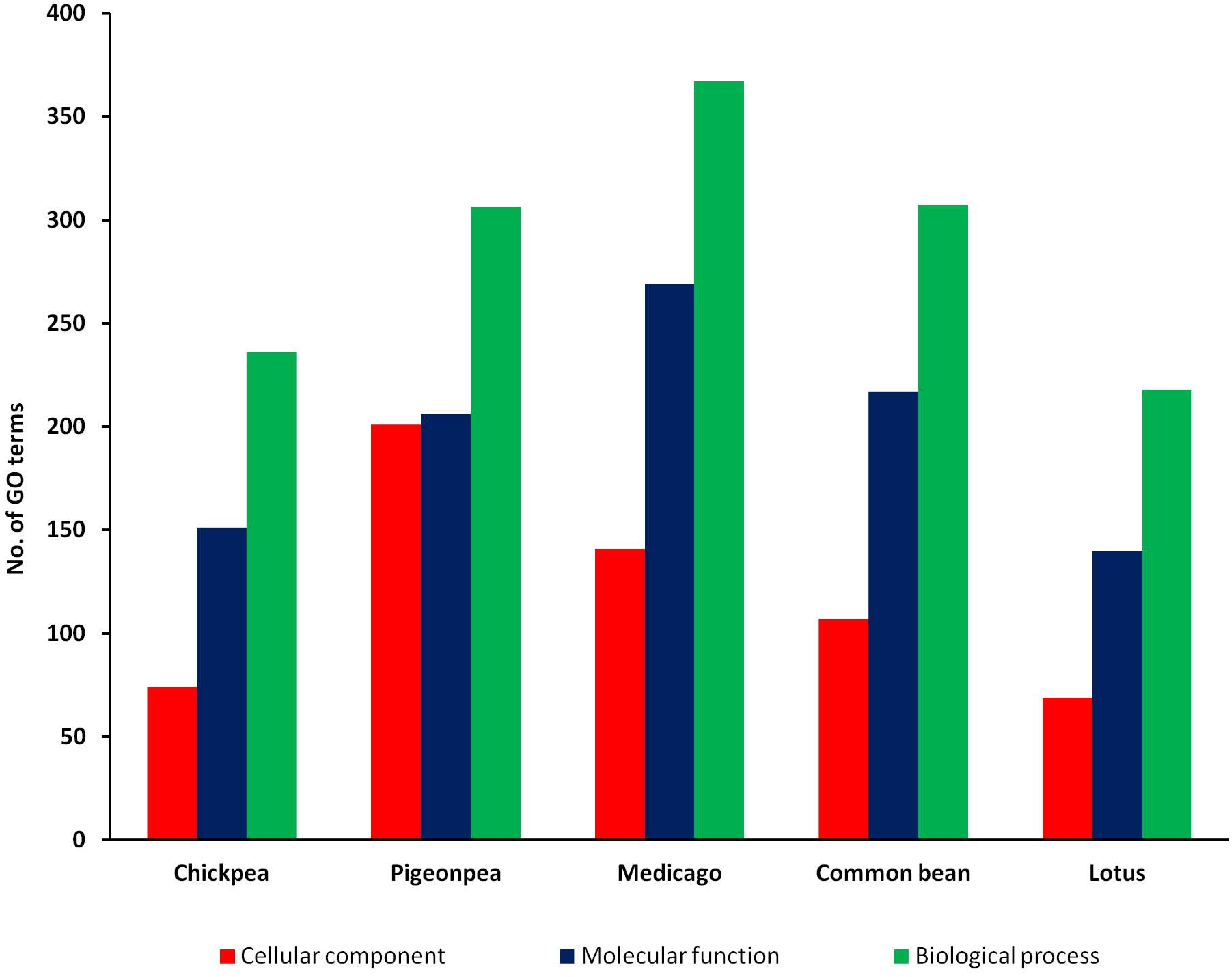

Supplementary Figure 16. Gene ontology assignment to the AP2/ERF sequences identified in the five legumes.
