# Supplementary material for: Genome‐wide dissection of AP2/ERF and HSP90 gene families in five legumes and expression profiles in chickpea and pigeonpea
Source: Plant Biotechnol J. 2016 Jan 23;14(7):1563–77. doi: 10.1111/pbi.12520 (PMC5066796; doi:10.1111/pbi.12520)
Supplement: Supplementary file 18 — Appendix S1 Experimental procedures, results and discussion. [file PBI-14-1563-s014.doc]

**Supplementary information**

**Experimental procedures**

**Identification of AP2/ERF and HSP90 proteins from five legume proteomes**

The latest versions of chickpea ([http://www.icrisat.org/gt-bt/ICGGC](http://www.icrisat.org/gt-bt/ICGGC/GenomeManuscript.htm)), pigeonpea ([http://www.icrisat.org/gt-bt/iipg/](http://www.icrisat.org/gt-bt/iipg/Genome_Manuscript.html)), Medicago (<ftp://ftp.jgi-psf.org/pub/compgen/phytozome/v9.0/Mtruncatula/annotation/>), Lotus ([ftp://ftp.kazusa.or.jp/pub/Lotus/Lotus_r2.5/](ftp://ftp.kazusa.or.jp/pub/lotus/lotus_r2.5/)) and common bean (<ftp://ftp.jgi-psf.org/pub/compgen/phytozome/v9.0/Pvulgaris/annotation/>) proteomes were downloaded from the respective sites. The AP2/ERF gene family sets from Arabidopsis and rice were retrieved from the Arabidopsis information resource, TAIR ([http://www.Arabidopsis.org/tools/bulk/sequences/index.jsp](http://www.arabidopsis.org/tools/bulk/sequences/index.jsp)) and RGAP (<http://rice.plantbiology.msu.edu/downloads_gad.shtml>). Sequence alignment was done using clustalw with default parameters considering full length protein sequences. Domains were checked using the respective peptide sequences in SMART database (<http://smart.embl-heidelberg.de/>) (Letunic et al., 2012).

**Composition, physio-chemical properties and annotation of AP2/ERF and HSP90 genes and proteins**

The gene structure was drawn using the Gene Structure Display Server (<http://gsds.cbi.pku.edu.cn/>). The isoelectric point (pI) was predicted using the Compute pI/Mw software (<http://www.expasy.ch/tools/pi_tool.html>). The sub-cellular localization was predicted using PSORT (<http://psort.hgc.jp/>) (Horton et al., 2007). The AP2/ERF sequences were annotated using Blast2GO (Conesa et al., 2005)

**Results and discussion**

The iso-electric points ranged from 4.23 to 11.41, protein length varied from 56 to 689 amino acids and the molecular weights strayed from 7.66 to 90.403 kDa among the five legumes (Supplementary Table 1-5). Chickpea showed minimum length (128 - 686 aa) and weight (7.66 kDa - 1.46 kDa) variation, whereas, Medicago (56 - 689; 6.85 kDa – 76.55 kDa) and lotus (51 - 818; 5.96 – 940.40 kDa) showed maximum variations. Such biochemical variations in members of same family strongly recommends the presence of putative novel variants and are in accordance with findings in foxtail millet (Lata et al., 2014).

**Conserved amino acids in AP2, ERF and DREB proteins**

Protein sequences of the two AP2 domains present in a AP2 protein, were found to have following conserved amino acid residues in most of the sequences: 3R, 4G, 5V, 9R, 11T, 12G, 13R, 15E, 17H, 19W, 20D, 32G, 43A, 44A, 47Y, 48D, 50A in the first AP2 domain and 2Y, 3R, 4G, 5V, 9H, 12G, 13R, 14W, 16A, 17R, 19G, 23G, 25K, 28Y, 29L, 30G, 37E, 38A, 39A, 41A, 42Y, 43D, 45A, 46A, 47I, 51G, 54A, 55V, 56T, 57N, 58F in the second domain in all five legumes. A linker of 30 amino acids between the two AP2 domains was also found to be conserved across all five legumes. Further, histidine was conserved in both AP2 domains of AP2 proteins. However, no histidine residue was conserved in ERF proteins. Threonine was found to be conserved in most of the AP2 proteins at position 6, which in case of ERF was arginine. Intriguingly, it was observed that in RAVs, glycine was found instead of valine, and alanine was found conserved at position 14 in AP2/ERF proteins. Conserved amino acids, VAEIRE in DREB subfamily and AAEIRD in ERF subfamily distinguished the two from each other.

More than 90% of DREB and ERF members in each of the 5 legumes studied contained the conserved amino acids namely:- 4G, 5V, 6R, 8R, 11G, 12K, 13W, 14V, 16E, 18R, 19E, 32R, 34W, 35L, 36G, 47A, 49D, 63N, 64F and 4G, 5V, 6R, 11G, 14A, 16E, 17I, 19D, 34W, 35L, 36G, 47A, 49D, 63N, 64F.

**Phylogeny of AP2/ERF proteins of Medicago, common bean and Lotus**

In Medicago, a total of 13 groups (Group I to XIII) were identified; Group I to IV composed of DREBs, Groups V to XI composed of ERFs; Group XII had both ERFs and 3 RAVs in distinct sub-clusters. Group XIII consisted of the AP2 proteins, with 7 ANT proteins clustered together (Supplementary Figure 7). In common bean, 14 such groups (I to XIV) were identified with group I to V constituting DREBs, groups VI to XII composed of ERFs, group XIII composed of RAVs and one soloist (Pv 008G131500) as an outlier, and group XIV contained the AP2 family which consisted of 10 ANT members (Supplementary Figure 8). AP2/ERF members in Lotus were categorized into 15 groups (I to XV) with groups I to V composing of DREB subfamily, groups VI to XIII containing ERFs including two RAVs clustered separately with group XI, group XIV having AP2 proteins with 3 ANT proteins, and group XV containing two soloists (Supplementary Figure 9). No soloist was identified in common bean.

**References**

Conesa, A., Gotz, S., Garcia-Gomez, J.M., Terol, J., Talon, M. and Robles, M. (2005) Blast2GO: a universal tool for annotation, visualization and analysis in functional genomics research. *Bioinformatics* **21**, 3674-3676.

Horton, P., Park, K.-J., Obayashi, T., Fujita, N., Harada, H., Adams-Collier, C. and Nakai, K. (2007) WoLF PSORT: protein localization predictor. *Nucleic Acids Res.* **35**, W585-W587.

Lata, C., Mishra, A.K., Muthamilarasan, M., Bonthala, V.S., Khan, Y. and Prasad, M. (2014) Genome-wide investigation and expression profiling of AP2/ERF transcription factor superfamily in foxtail millet (*Setaria italica* L.). PLoS ONE, 9, e113092.

Letunic, I., Doerks, T. and Bork, P. (2012) SMART 7: recent updates to the protein domain annotation resource. *Nucleic Acids Res.* **40**, D302-305.
